# Reframing gene essentiality in terms of adaptive flexibility

**DOI:** 10.1186/s12918-018-0653-z

**Published:** 2018-12-17

**Authors:** Gabriela I. Guzmán, Connor A. Olson, Ying Hefner, Patrick V. Phaneuf, Edward Catoiu, Lais B. Crepaldi, Lucas Goldschmidt Micas, Bernhard O. Palsson, Adam M. Feist

**Affiliations:** 1 Department of Bioengineering, University of California, San Diego, La Jolla, 92093 CA USA; 2 Department of Chemical Engineering, University of Ribeirão Preto, São Paulo, Brazil; 3 Department of Chemical and Petroleum Engineering, Fluminense Federal University, Niterói, Rio de Janeiro, Brazil; 4 Novo Nordisk Foundation Center for Biosustainability, Technical University of Denmark, Lyngby, Denmark; 5 Department of Pediatrics, University of California, San Diego, La Jolla, 92093 CA USA; 6 Department of Bioinformatics and Systems Biology, University of California, San Diego, 92093 La Jolla, CA USA

**Keywords:** Essentiality, Genome-scale model, Adaptive evolution

## Abstract

**Background:**

Essentiality assays are important tools commonly utilized for the discovery of gene functions. Growth/no growth screens of single gene knockout strain collections are also often utilized to test the predictive power of genome-scale models. False positive predictions occur when computational analysis predicts a gene to be non-essential, however experimental screens deem the gene to be essential. One explanation for this inconsistency is that the model contains the wrong information, possibly an incorrectly annotated alternative pathway or isozyme reaction. Inconsistencies could also be attributed to experimental limitations, such as growth tests with arbitrary time cut-offs. The focus of this study was to resolve such inconsistencies to better understand isozyme activities and gene essentiality.

**Results:**

In this study, we explored the definition of conditional essentiality from a phenotypic and genomic perspective. Gene-deletion strains associated with false positive predictions of gene essentiality on defined minimal medium for *Escherichia coli* were targeted for extended growth tests followed by population sequencing and transcriptome analysis. Of the twenty false positive strains available and confirmed from the Keio single gene knock-out collection, 11 strains were shown to grow with longer incubation periods making these actual true positives. These strains grew reproducibly with a diverse range of growth phenotypes. The lag phase observed for these strains ranged from less than one day to more than 7 days. It was found that 9 out of 11 of the false positive strains that grew acquired mutations in at least one replicate experiment and the types of mutations ranged from SNPs and small indels associated with regulatory or metabolic elements to large regions of genome duplication. Comparison of the detected adaptive mutations, modeling predictions of alternate pathways and isozymes, and transcriptome analysis of KO strains suggested agreement for the observed growth phenotype for 6 out of the 9 cases where mutations were observed.

**Conclusions:**

Longer-term growth experiments followed by whole genome sequencing and transcriptome analysis can provide a better understanding of conditional gene essentiality and mechanisms of adaptation to such perturbations. Compensatory mutations are largely reproducible mechanisms and are in agreement with genome-scale modeling predictions to loss of function gene deletion events.

**Electronic supplementary material:**

The online version of this article (10.1186/s12918-018-0653-z) contains supplementary material, which is available to authorized users.

## Background

The term essential has been used to define those components of the cell that are required to sustain cell growth. Defining the essential components of life in organisms large and small has been a topic of great scientific interest, and, on one extreme, there is a growing effort to understand the basic principles of life by studying and even synthesizing minimal organisms [1, 2]. Beyond understanding the basic genotype-phenotype connection of life, studies of gene essentiality provide knowledge for medical and industrial applications. Essential genes provide targets for antibacterial drug discovery. For example, by carefully targeting an essential cell component in a bacterium as virulent as *Mycobacterium tuberculosis*, we can strive to treat pathogenic bacterial infections in a targeted and rational manner while at the same time avoiding harmful side-effects to the host organism [3]. Strategic, logical drug design has become increasingly important in the face of rising numbers of antibiotic resistant pathogens [4–6]. Thus, research studies of gene essentiality have become important knowledge sources for the advancement of science and medicine.

How do we go about defining the set of essential genes for an organism? Increased availability of genome sequences has led to detailed experimental and computational examination of gene functions at a genome scale in several model organisms, including *E. coli*. A clear method for studying gene essentiality is the systematic experimental disruption of genes. One such study resulted in a collection of 3985 single-gene deletion strains for the K-12 derivative strain of *E. coli* BW25113 [7]. Other studies have utilized high-throughput transposon mutagenesis as a tool for gene disruption and identification of all essential genome elements beyond protein-coding sequences [8]. Beyond experimentally defining essential genes, computational tools such as constraint-based modeling have been used for predicting essential components of cells, most applicably metabolic components [9].

Expanding knowledge of the cellular components contributing to metabolism has allowed for the construction of genome-scale models of metabolism. The comprehensive metabolic model for *E. coli*, *i*JO1366 contains information related to 1366 metabolic genes and their associated 2251 reactions. Such models can be used to study bacteria from a whole-cell, systems biology perspective [10–12]. By removing genes from the model and performing flux-balance analysis, predictions about gene essentiality on defined growth media can be made. These predictions can then be compared to experimental data and provide insight into existing knowledge gaps when inconsistencies are encountered [13]. In general, such metabolic models have shown a high degree of accuracy in predicting the effect of gene knockouts. The *i*JO1366 reconstruction showed an 89.8% accuracy in predicting gene knockout growth/no growth behavior [10] and its subsequent update the *i*ML1515 reconstruction showed a 93.4% accuracy [12]. Failure modes are referred to as false negative or false positive predictions. False negative predictions are inconsistencies that occur when a model predicts a gene to be essential, but experiments show the gene to be non-essential. This can occur when there is missing knowledge regarding alternate pathways or isozymes, as has been previously demonstrated [14]. False positive (FP) predictions are inconsistencies that occur when the model predicts a gene to be non-essential, but experiments show the gene to be essential. Such instances can be attributed to the inclusion of unrealistic reactions in the model. They can also, however, be attributed to flaws in the experimental data. For example, high-throughput growth screens conducted in plate format are often stopped after 24 or 48 h of growth [7, 15, 16]. These screens might not capture those strains that are slower to grow. Furthermore, it is also possible that the models predict growth that is not feasible without some form of genetic change or adaptation, which adjusts regulation of predicted growth enabling pathways. High-throughput screens are rarely followed by whole genome sequencing given the assumption that mutations are not accrued in such a short period of time. Thus, growth that is accompanied by genetic change is not captured by such growth screens.

Essentiality is widely accepted to be conditional [17, 18]. Although there is a large set of non-metabolic and metabolic genes that are essential regardless of growth environment (e.g., genes related to replication, transcription, translation, co-factor biosynthesis, and cell structure), many genes essential for growth in one environment might not be essential in another, given a different nutrient composition. However, essentiality may also be discussed in evolutionary terms. Upon the removal of an essential gene, it is possible that a short period of adaptation is sufficient to activate a redundant pathway or isozyme and enable growth. On the other hand, some genes may be essential regardless of whether or not an adaptive period is provided. Thus, we can also consider a spectrum of essentiality that is related to adaptability. This has been discussed and demonstrated in studies of multi-copy suppression and adaptive laboratory evolution [14, 19]. The extent of redundant pathways in *E. coli* is yet to be fully elucidated; however, underground metabolism and enzyme promiscuity have been shown to play critical roles in adaptation to new growth environments or in response to genetic perturbation [14, 20, 21].

In this study, we utilize previously reported FP predictions of essentiality [13] to identify gene-deletion strains that may be considered ‘non-essential’ given a longer incubation period. This study examines gene-deletion strains which can be isolated in pure culture in vitro and categorizes them into three categories (expanding on definitions previously used [17, 18]). First, if a knockout (KO) strain cannot grow on a defined medium where the wild type strain can grow, ‘conditional essentiality’ is established. Second, if a KO strain is able to grow on the defined medium where the wild type strain can also grow, ‘non-essentiality’ is established. Third, if a KO strain does not initially grow on a defined medium, but is able to grow given an adaptive period and the acquisition of mutations, ‘non-essentiality with mutations’ is established. Longer growth tests are followed by whole genome sequencing, transcriptome analysis of selected strains, and interpretation of any resulting mutations and expression changes to determine the adaptive mechanisms required for the rescue of the KO strains analyzed. The results presented demonstrate a striking agreement between model-predicted alternate isozymes/pathways and observed mutations and shed light on the dynamics of growth observed for various non-essential genes.

## Results

### Identifying gene targets

**Fig. 1:**
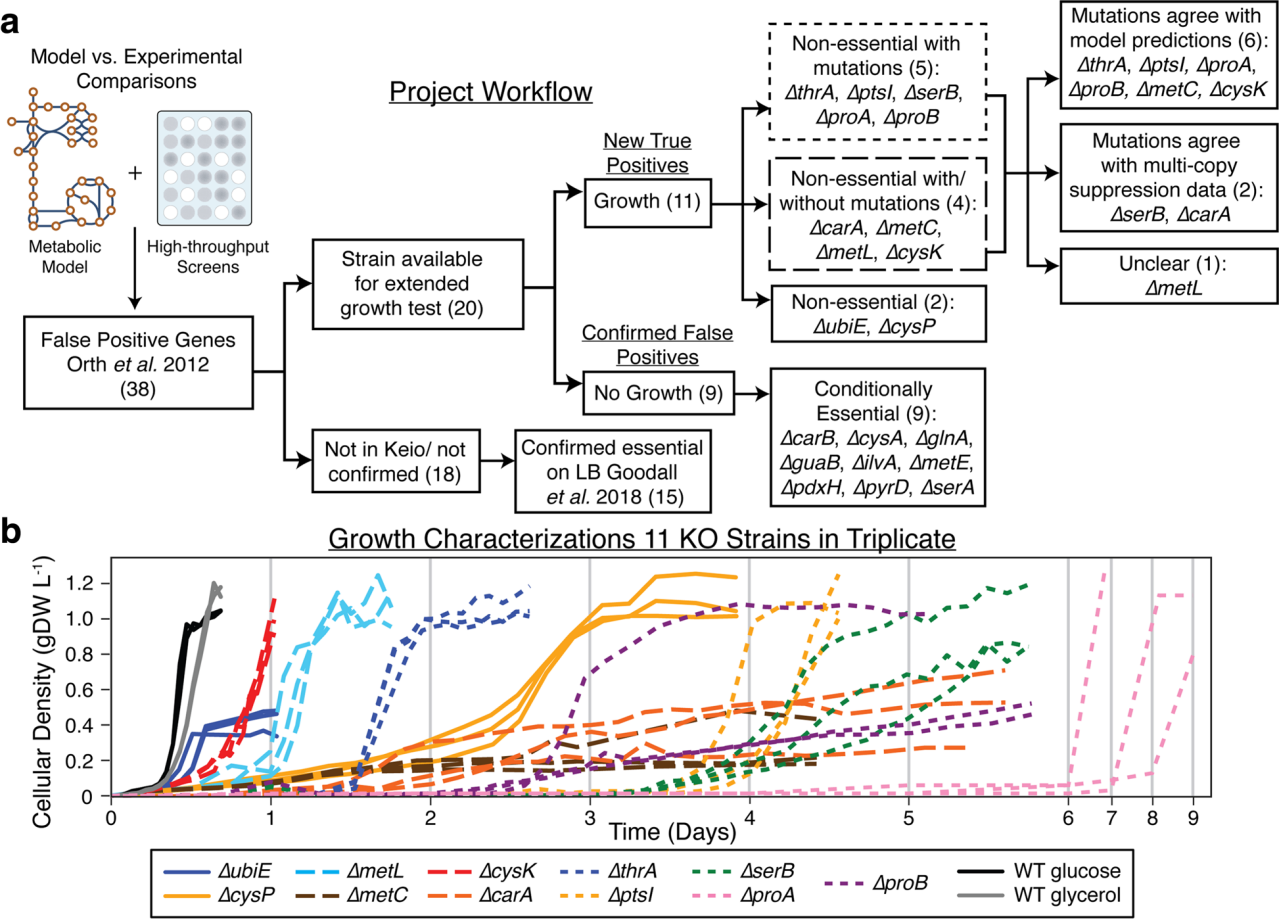
Project workflow and growth characterizations of false positive strains. **a** A workflow summarizing the sequence of analyses followed and results from this study. A Keio gene KO collection strain was not available for approximately half of the corresponding false positive genes listed in [13], most likely due to essentiality on rich growth media [22]. Of those strains that were viable in rich media and thus available for a longer growth test, approximately half showed growth in minimal defined media. Those strains that grew were analyzed for mutations. Five false positive strains showed mutations in all replicate experiments sequenced. Four strains showed mixed results, meaning that only some populations accrued mutations. Two strains showed no mutations in any replicate samples. The nine strains that showed mutations in at least some populations were further analyzed in the context of model-predicted alternate pathways and historical data. Six of these cases showed agreement with model-predictions, two showed agreement with previous reports of multi-copy suppression [81], and mutation analysis for one case was not clearly linked to either. **b** Growth curves of eleven Keio collection strains associated with false positive predictions in defined minimal media. Growth data in terms of cellular density in grams of dry weight per Liter (gDW/L) is reported for the FP gene KO strains. Those strains that accrued mutations in all replicate populations during this growth test are noted with small dashed lines. Those strains that showed mixed results, showing mutations in only some populations, are noted with larger dashed lines. All Keio strains were grown in M9 minimal medium with glucose as the carbon source with the exception of *ΔcysK* and *ΔcysP* which utilized a glycerol carbon source. Growth of the wild type strain in glucose and glycerol is also provided as a point of reference (black and grey growth curves)

The genes explored in this study were chosen because of essentiality discrepancies observed between *in silico* predictions and in vivo observations [13]. Such discrepancies indicate areas for discovery or better understanding as they point out differences between in vivo screens and computations based on the collected wealth of knowledge for a given organism [9]. These discrepancies were previously identified as FP model predictions (instances where the model predicts that a gene is non-essential, but experimental studies have identified the gene as essential in the particular growth environment). FP model predictions are believed to occur due to the inclusion of a ‘non-physiological’ model reaction such as an unrealistic alternate isozyme or pathway. On the other hand, FP predictions may also occur if there are errors in experimental calls of essentiality. It is possible that short-term high-throughput growth screens could result in genes being identified as essential when in actuality they require more incubation time to display growth. In these cases, it is possible that the metabolic model annotation of an alternate pathway or isozyme is correct and such a predicted alternate pathways might not be sufficiently expressed prior to a period of adaptation. This study examined the possibility that FP predictions are caused by experimental limitations. Furthermore, the dynamics and mechanisms behind a cell’s ability to still display reproducible and robust growth during time frames longer than normal wild-type growth profiles were also examined. This was accomplished by performing more extensive growth analysis of FP genes identified for the *E. coli* metabolic model, *i*JO1366. 38 genes previously identified as FP predictions [13] were utilized as a starting point for this study (Fig. 1a, Additional file 1). These were genes associated with FP predictions in defined minimal media conditions.

### Growth screens considering longer time scales

Extended growth tests were performed on FP associated gene-deletion strains with the hypothesis that model-predicted alternate pathways would rescue growth given longer incubation. Of the 38 FP associated gene KO strains identified as potential targets for long growth incubations, 20 were available in the Keio collection and confirmed by PCR (polymerase chain reaction) (Additional file 1). Comparison of 18 genes for which no Keio strains existed to an essential gene dataset reported by a study conducting transposon-directed insertion site sequencing [22], confirmed that 15 of the unavailable Keio strains were on account of gene essentiality on nutrient-rich media (Additional file 1, Fig. 1a). Therefore, such gene KO strains are very unlikely to be constructed by the approach used in the Keio collection or any KO method and these unavailable strains were not considered for the longer growth tests explored in this study [7] (Fig. 1a). The 20 confirmed gene-deletion strains available (Additional file 1) were grown in a pure culture rich nutrient undefined medium (Luria-Bertani LB broth) and then used to inoculate minimal medium for the extended growth test. Growth of these 20 gene-deletion strains was monitored periodically over the course of approximately two weeks or until growth was observed. If growth was observed, the culture was passed to fresh media to ensure that the growth would persist and was not a by-product of residual LB media. Nine strains did not grow during the extended growth test and were classified as conditionally essential (not essential in LB, but essential for growth in the minimal medium tested) (Fig. 1a, Additional file 1). For the KO strains that did display growth, such long incubation growth tests showed that eleven of the twenty previously identified essential genes were actually non-essential (Fig. 1, Table 1). To confirm the reproducibility of these results, these eleven gene-deletion strains were grown again in triplicate (with some strains tested more than triplicates, up to nine biological replicates) and their cell density monitored more closely to acquire a more detailed view of their growth trajectories (Fig. 1b). These eleven gene KO strains were the main focus of this work.

**Table 1:** Strain details from growth characterizations

Keio strain	Possible alternate	Final cell density (gDW/L)	Time to > half	Mutations
	genes/pathways	*mean, Std. Dev., %RSD	final density (Hrs)	flask1
			*mean, Std. Dev., %RSD	population?
WT glucose	-	1.0, <0.01, 0.2%	10, <1, <0.01%	No
WT glycerol	-	1.1, 0.04, 3%	12, <1, 0.04%	No
*Δ* *ubiE*	Alternate growth using	0.43, 0.08, 20%	14, 1, 9%	No
	demethylmenaquinone^1^			
*Δ* *cysK*	*cysM* ^2^	1.0, 0.1, 10%	21, <1, <0.01%	Variable
*Δ* *metL*	(*thrA* or *lysC*)^2^	1.0, 0.1, 9%	28, 2, 7%	Variable
*Δ* *metC*	(*tnaA* or *malY*)^2^,	0.28, 0.1, 50%	36, 9, 20%	Variable
	(*malY*, *alr*, *fimE*)^3^			
*Δ* *thrA*	(*metL* or *lysC*)^2^	1.1, 0.1, 8%	43, <1, 0.04%	Yes
*Δ* *carA*	(*yahI* or *arcC* or *yqeA*)^2^,	0.50, 0.2, 40%	58, 17, 30%	Variable
	(*carB*, *ygiT*, *cho*, *yncK*)^3^			
*Δ* *cysP*	(*modA* + *modB* + *modC*)^2^	1.1, 0.1, 10%	65, <1, 0.2%	No
*Δ* *proA*	*argE* ^2^	1.1, 0.3, 20%	190, 26, 10%	Yes
*Δ* *proB*	*argE* ^2^	0.66, 0.3, 50%	83, 10, 10%	Yes
*Δ* *ptsI*	*galP*^2^, (*fucP*, *xylE*, *galE*)^3^	1.1, 0.1, 10%	100, 5, 5%	Yes
*Δ* *serB*	*glyA*^2^, (*gph*, *hisB*, *ytjC*)^3^	0.95, 0.2, 20%	110, 8, 7%	Yes

^1^Evidence for this described in [64]. ^2^*In silico* prediction, *i*JO1366 genome-scale model of metabolism [13]. ^3^Experimental multicopy suppression evidence [19]. *The data used from triplicate experiments represented in Fig. 1b was used to calculate means, standard deviations (St. Dev.), and percent relative standard deviations (%RSD)

Growth experiments showed a great deal of fitness diversity among the eleven gene-deletion strains (Fig. 1b, Table 1). While four of the eleven KO strains (*ΔcysK*, *ΔmetL*, *ΔthrA*, *ΔubiE*) showed reproducible growth to their respective final cellular densities within the first 48 h of incubation, the remaining strains displayed more variability in the time necessary to display growth. Furthermore, some replicates showed a range in growth dynamics between replicate experiments, which is reflected in the standard deviation of the mean time required to reach at least half of the final density observed during these growth tests (Table 1, Additional file 2). For example, *ΔproB* experiments showed a high degree of variability between the three replicates tested. One replicate reached its final density, near that of wild-type, around Day 4 whereas the other two replicates had reached half this level around Day 6 (Fig. 1b). Several gene-deletion strains also showed variability in the final density achieved. While several strains such as *ΔthrA*, *ΔcysK*, and *ΔmetL* showed typical growth trajectories similar to the wild type strain (with longer lag phases), other strains such as *ΔcarA*, *ΔmetC*, and *ΔubiE* displayed significantly slower growth rates and reached approximately half of the final cell density observed for the wild type during the testing period. We hypothesized that the diverse range of growth phenotypes observed could be attributed to differences in adaptive mechanisms required for growth. This was further studied by examining mutations acquired during the growth experiments.

### Mutation analysis driven by parallel evolution

The guiding principle for mutation analysis was to identify evidence of parallelism between replicate experiments at the level of genes mutated to determine likely mechanisms of adaptation. Parallel evolution at the gene-level has been demonstrated to provide compelling evidence specific to applied selection pressures [23, 24] or, for this study, in response to genetic perturbations. Parallelism was examined by first identifying key mutation events across replicate experiments, such as multiple unique mutations occurring within the same gene or multiple unique mutations in linked metabolic genes and their regulatory elements (Table 2). The identification of even a single mutation shared between two samples (i.e., replicate experiments) at the gene level is highly unlikely (Fisher’s exact test *p*-value < 0.005, Additional file 3). These key mutation events were interpreted in the context of model-predicted alternate isozymes and pathways or other experimental studies to further frame potential adaptive evolution events. Model-associated isozymes were identified by examining model gene-protein-reaction associations and model-associated alternate pathways were identified by examining those reactions associated with alternate growth solutions (Table 1).

**Table 2:** Flask 1 population mutations

Keio	Exp. #	Fraction	Gene	Protein change	Perceived impact
strain		population			
*Δ* *thrA*	4	0.58	*metK*	N226S	-
	2	0.46	*metK*	G235A	-
	2	0.23	*metJ*	V46E	*Reduce MetJ repression^1^
	1	0.79	*metJ*/*metB*	Intergenic (-211/-66)	*Reduce MetJ repression^1^
	3	-	117 genes	130 kbp, 1.2X GDA	Increase *metL* expression
			[*rrlA*-*rrfB*]		
*Δ* *ptsI*	4, 5	1.0, 0.82	*ydhF*	G65S	-
	(1, 4,	(1.0, 1.0,	*metK*/*galP*	Intergenic (+328/-96)	*Reduce GalR repression^2^
	5, 6)	0.36, 0.39)			
	5	0.25	*metK*/*galP*	Intergenic (+333/-91)	Reduce GalR repression^2^
	3	1.0	*metK*/*galP*	Intergenic (+334/-90)	Reduce GalR repression^2^
	6	0.57	*metK*/*galP*	Intergenic (+339/-85)	Reduce GalR repression^2^
	3	1.0	*crp*	T141P	Global regulatory effects^3^
	5	0.70	*crp*	G142S	Global regulatory effects^3^
	6	0.59	*crp*	G142D	Global regulatory effects^3^
	4	1.0	*crp*	R143H	Global regulatory effects^3^
	1	1.0	*crp*	A145V	Global regulatory effects^3^
	6	0.43	*crp*	I187T	Global regulatory effects^3^
	7	1.0	96 genes	99 kbp, 2X GDA	Affect *cyaA* expression
			[*rrsC*-*rrlA*]		
*Δ* *serB*	1	0.67	*hisL*/*hisG*	Intergenic (+41/-105)	*Increase *his* operon expression^4^
	5	0.43	*yhaC*/*garK*	Intergenic (+653/+370)	-
	1	0.27	*yhaC*/*garK*	Intergenic (+883/+140)	-
	6	0.88	*hisR*	His tRNA (5/77 bp)	Increase *his* operon expression^4^
	2	0.82	*hisR*	His tRNA (48/77 bp)	Increase *his* operon expression^4^
	4	0.92	*hisR*	His tRNA (67/77 bp)	*Increase *his* operon expression^4^
	7	0.71	*hisR*	His tRNA (72/77 bp)	Increase *his* operon expression^4^
*Δ* *proA*	2	1.0	*proB*	1 bp Del	-
	1	0.77	*argD*	G282D	Reduce ArgD activity
	2	0.56	*argD*	Del (772-774/1221 bp)	Reduce ArgD activity
	4	0.43	*argD*	Q154*	Reduce ArgD activity
	3	0.34	*argD*	G49R	Reduce ArgD activity
*Δ* *proB*	6, 7	0.83, 0.72	*glnA*	F463L	Reduce GlnA activity
	5	0.86	*glnA*	D187E	Reduce GlnA activity
	4	0.45	*glnA*	G179C	Reduce GlnA activity
	4	0.23	*glnA*	H172R	Reduce GlnA activity
	1	1.0	*glnA*	G171S	Reduce GlnA activity
	2	0.86	*glnA*	E156D	Reduce GlnA activity
	3	1.0	*glnA*	S148F	Reduce GlnA activity
*Δ* *carA*	7	0.27	*carA*/ *carB*	Intergenic (+2/-16)	Increase *carB* expression
	5	0.86	*carB*	L11L	-
	3, 7	0.31, 0.81	*rpoS*	E96*	-
	3, 7	1.0	508 genes	520 kbp, 2X GDA	Increase *carB* expression
			[*insD6*-*insD1*]		
*Δ* *metC*	4	0.55	*malI*	10bp Dup (227/1029 bp)	Reduce MalI activity^5^
	2	0.32	*malI*	Q55*	Reduce MalI activity^5^
	3	0.55	*malX*	Q529Q	-
*Δ* *metL*	2, 4	0.72, 0.38	*rpoS*	L317R	-
	4	1.0	*metB*	1 bp Del (1144/1161 bp)	-
*Δ* *cysK*	1	1.0	2,062 genes	2.1 Mbp, 2X GDA	Increase *cysM* expression
			[*insL3*-*insL1*]		
	3	0.21	*sspA*/*rpsI*	Intergenic (-10/+385)	-

GDA abbreviation stands for genome duplication amplification event. ^1^Binding site recognition sites predicted in [26]. ^2^Evidence to support binding site in [31]. ^3^Information regarding CRP global regulatory effects in [32–34]. ^4^Attenuator-model of regulation for histidine operon described in [44–47]. ^5^Information related to the MalI transcriptional repressor in [38, 39]. *Perceived impact was further confirmed by RNA sequencing results (Fig. 3, Table 3)

**Table 3:** Differential expression of alternate pathways/genes in knockout strains compared to wild type (RNAseq Data)

Keio strain	Experiment *#*, flask *#*,	Predicted	Log_2_(Fold Change)	P-adjusted value
	clone/population	alternate gene	experiment vs. wild type	
*Δ* *thrA*	Exp.1, Flask 5, Clone	*metL*	2.18	2.06E-7
*Δ* *thrA*	Exp.2, Flask 1, Population	*metL*	1.13	3.48E-2
*Δ* *ptsI*	Exp.1, Flask 5, Clone	*galP*	1.73	2.50E-2
*Δ* *ptsI*	Exp.3, Flask 1, Population	*galP*	0.908	5.46E-1
*Δ* *serB*	Exp.1, Flask 5, Clone	*hisB*	3.75	3.60E-11
*Δ* *serB*	Exp.4, Flask 1, Population	*hisB*	3.28	2.36E-9

Following growth characterization experiments, genomic DNA was sequenced. Samples were taken from the first flask of growth in minimal medium and prepped for whole genome sequencing (referred to as flask 1 populations, see Materials and Methods) (Additional file 4). In addition to flask 1 population sample sequencing, the starting inoculation strain grown in nutrient-rich media was sampled and sequenced as a reference for mutation analysis (Additional file 5). It is of importance to note that four starting strains isolated from the Keio collection and grown in rich medium (*ΔcarA*, *ΔcysK*, *ΔmetC*, and *ΔptsI*) contained mutations prior to growth on the defined minimal medium (Additional file 5). These base mutations, possibly acquired during the construction of these strains, might have been selected for during growth on the nutrient rich medium and will be addressed later on a case-by-case basis. Of the eleven gene-deletion strains that grew during the long growth tests, two strains (*ΔcysP* and *ΔubiE*) did not reveal any prevalent mutations in the flask 1 populations that were sequenced and were thus considered non-essential and actually ‘True Positive’ model predictions (Fig. 1a). Five (*ΔthrA*, *ΔptsI*, *ΔserB*, *ΔproA*, and *ΔproB*) accrued prevalent mutations in all flask 1 populations sequenced (occurring at a fraction of the total population > 0.2 as determined by read-depth) that were not present in the inoculating cultures (Table 2). These strains that acquired mutations during growth were considered non-essential with mutations (Fig. 1a). Four strains (*ΔcysK*, *ΔmetC*, *ΔmetL*, and *ΔcarA*) showed mutations in some of the flask 1 populations sequenced and were considered non-essential with/without mutations since it appeared that it was possible to attain growth without mutations; however, it was possible that mutations were below the detection criteria (occurring at a fraction < 0.2) and the population is highly heterogeneous with many mutations or the mutational events are outside the scope of the computational mutation identification pipeline utilized (e.g., genome rearrangements). In summary, those strains that showed prevalent mutations in some or all population samples sequenced were considered non-essential with mutations, whereas those that showed no mutations were considered non-essential and actually True Positive predictions (Fig. 1a).

The nature of the mutations that were observed varied in terms of structural or regulatory mutations. Regulatory mutations observed included mutations in intergenic regions, transcription factors, tRNAs, as well as large regions of genome amplification. Mutations were considered structural if they occurred within the coding region of a metabolic gene. The following sections highlight the diversity and extent of parallel mutation events observed during these extended growth experiments.

### Mutation enrichment in genetic elements linked to predicted alternate pathways/isozymes

**Fig. 2:**
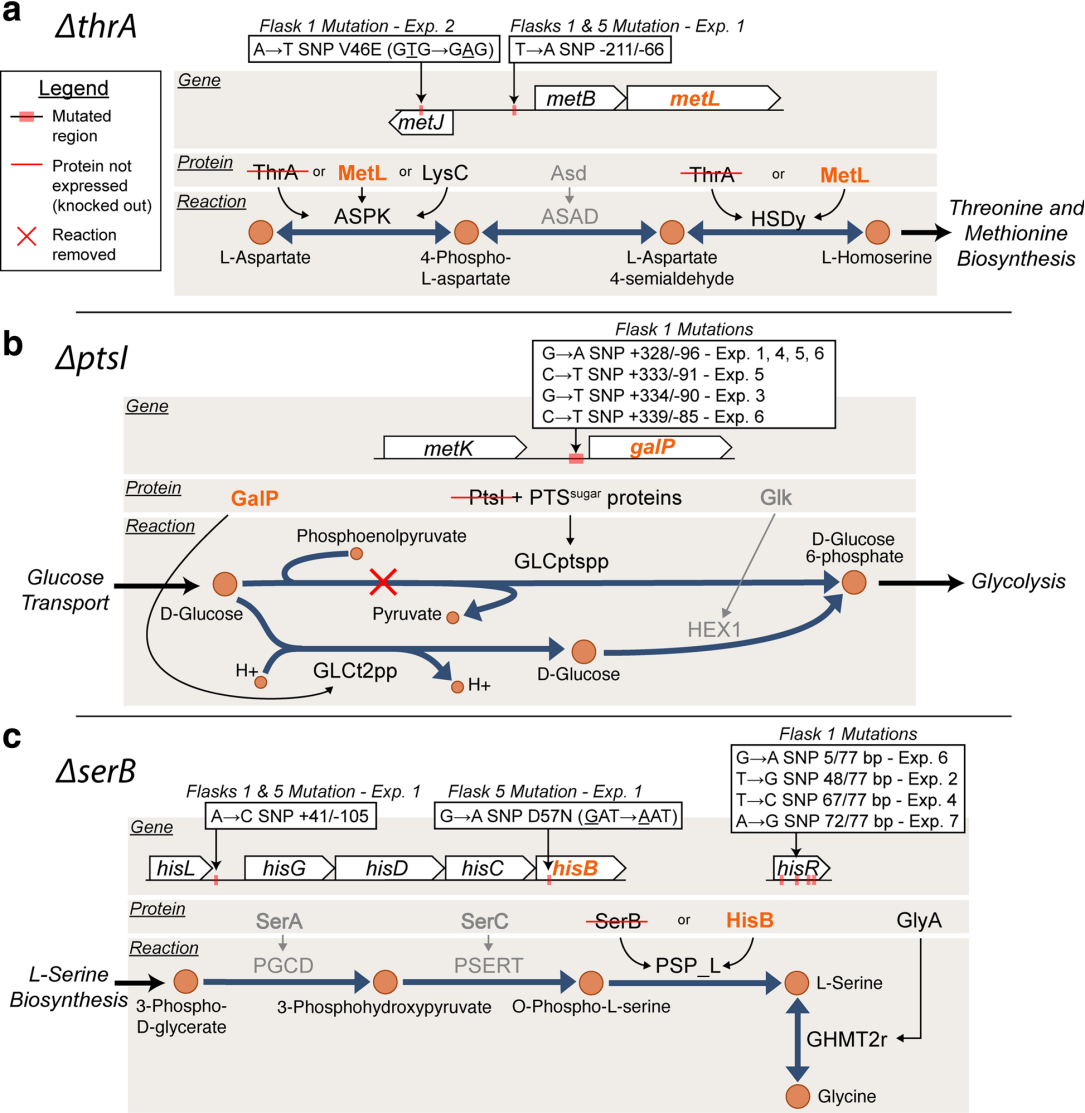
Pathway maps related to *ΔthrA*, *ΔptsI*, and *ΔserB* false positive cases. Whole genome sequencing analysis revealed that for two out of three of these cases the model prediction was in agreement with the observed utilized pathway as inferred from mutation analysis. The associated gene-protein-reaction information for each case is highlighted. In **a**, the mutation results for *ΔthrA* imply that MetL (highlighted in orange) is the enzyme responsible for the isozyme activity as predicted. **b** Results for *ΔptsI* suggest that the predicted alternate pathway related to GalP is utilized in the absence of PtsI. **c** Results for *ΔserB* suggest that contrary to the predicted GlyA associated alternate pathway, HisB is responsible for rescuing growth in the absence of SerB

**Fig. 3:**
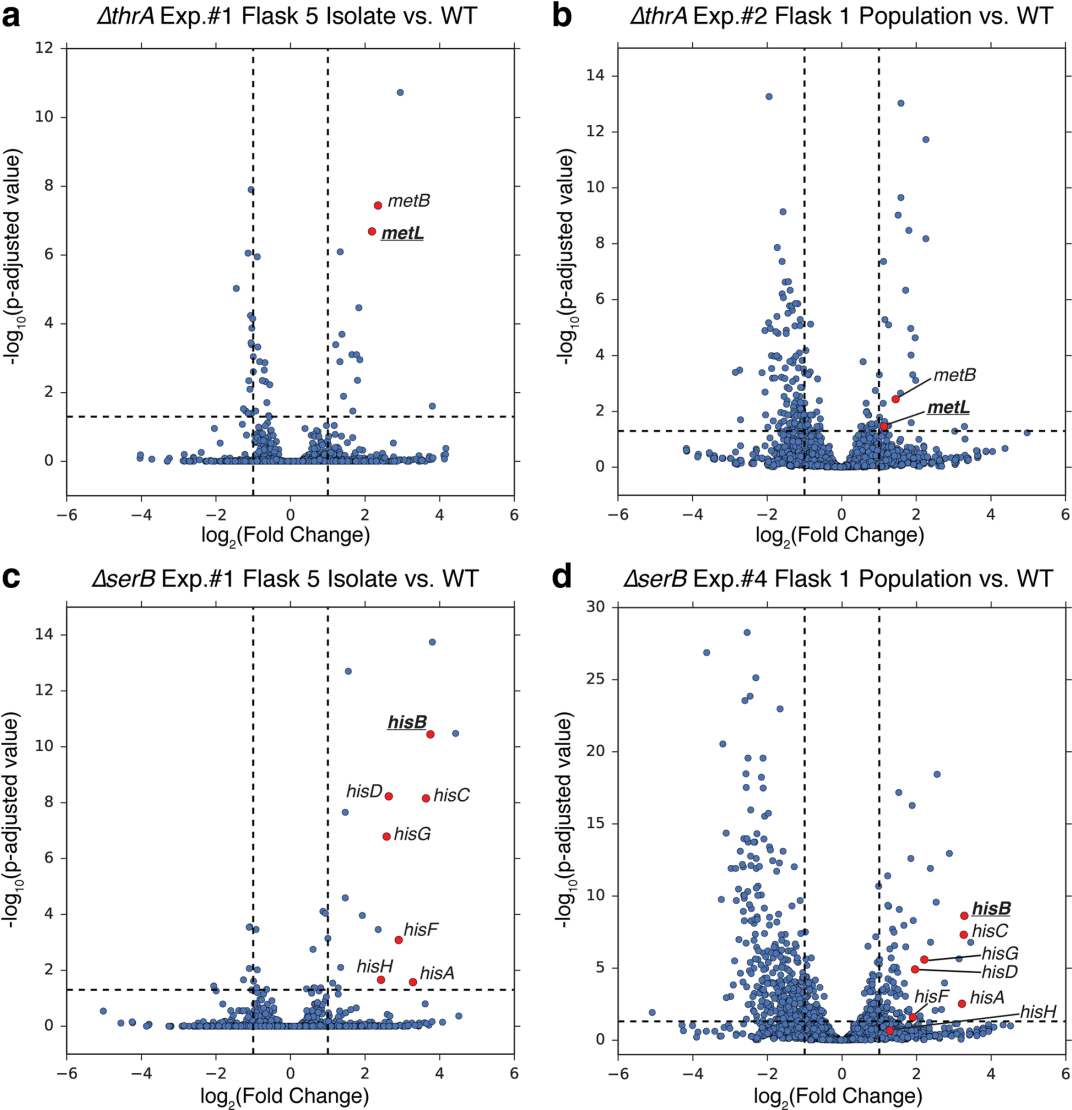
RNA sequencing data for *ΔthrA* and *ΔserB* experiments support isozyme predictions and mutation analysis. Volcano plots display log_2_(fold change) on the x-axis and -log_10_(p-adjusted value) on the y-axis for RNA sequencing data acquired for *ΔthrA* and *ΔserB* experiments. The log_2_(fold change) and p-adjusted value data was acquired comparing the experimental condition expression data (biological duplicate replicates) to wild type BW25113 expression data (biological duplicate replicates). Each dot in the plots corresponds to the differential expression data of a gene. Panels A. and B. correspond to the data for *ΔthrA* experiments and panels **c** and **d** correspond to the data for *ΔserB* experiments. The vertical dashed lines in each plot corresponds to the 2X fold change line in either direction (log_2_(fold change) = 1, -1) and the horizontal dashed line corresponds to the 0.05 p-adjusted value line (-log_10_(p-adjusted value) = 1.3). Highlighted in red in panels **a** and **b** are the *metL* and *metB* genes, which are expressed in the same operon. Of interest for the *thrA* experiments was the up-regulation of the predicted isozyme, *metL*. Highlighted in red in panels **c** and **d** are *his* operon genes that were up-regulated in both *ΔserB* experiments. Of particular interest was *hisB* expression, the predicted isozyme for *serB*

In order to elucidate the mechanism of adaptation for the FP gene-deletion strains, key mutations were analyzed in the context of model-predicted alternate pathways or isozymes (Tables 1 and 2). The first few cases highlighted were in excellent agreement with the model-predicted alternate functional pathway. For the *ΔthrA* and *ΔptsI* strains, mutations were enriched in intergenic regions that could be linked to model-predicted alternate isozymes (*metL*) or pathways (*galP*) (Fig. 2a, b). ThrA is annotated as a bifunctional aspartate kinase and homoserine dehydrogenase. The metabolic model for *E. coli*, *i*JO1366, lists MetL as an alternative bifunctional enzyme capable of catalyzing the same reactions [10], which is also supported by in vitro enzyme assays [25]. It was thus speculated that the intergenic mutations between *metJ* and *metB* (Fig. 2a) affect transcription of *metL* [26]. Furthermore, the mutation within the coding region of *metJ*, the transcriptional repressor for various *met* operon genes, was also proposed to influence expression of *metL*. Lastly, in another independent replicate, a genome duplication amplification was also detected (Table 2) which included the metL gene and was thus hypothesized to increase *metL* expression. RNA sequencing data demonstrated that the predicted compensating isozyme *metL* was indeed up-regulated in the adapted *ΔthrA* strains (Fig. 3a, b). The expression of *metL* was significantly up-regulated in the Exp. 1, Flask 5 clone assayed at an increase of 4.5X (log_2_(fold change) = 2.18) (Table 3, Fig. 3a). This supported the hypothesis that the *metJ*/*metB* intergenic mutation influenced expression of *metL* (Table 2, Fig. 3a). The expression of *metL* was also significantly up-regulated in the Exp. 2, Flask 1 population assayed at an increase of 2.2X (log_2_(fold change) = 1.13) compared to wild type (Table 3, Fig. 3b). This population assayed possessed an internal *metJ* mutation (Table 2, Fig. 3b). Together, the mutation enrichment analysis and RNA sequencing data supported the isozyme prediction made by the metabolic model.

Another FP case for which mutations showed strong agreement with model-predictions was *ptsI* (Fig. 2b). PtsI is part of the well-characterized phosphoenolpyruvate:sugar phosphotransferase system (PTS^sugar^) [27–29]. This system is responsible for the phosphorylation and transport of various carbohydrate substrates including glucose; however, *E. coli* contains an alternative system for glucose transport and phosphorylation linked to *galP* in *i*JO1366 (Fig. 2b). GalP is a proton symporter involved in galactose transport, but it has also been shown to transport glucose in *ptsG* and *ptsM* mutants [30]. The mutation evidence observed in the *ΔptsI* experiments in this study was suggestive of D-glucose transport via the *galP* alternate pathway, as predicted by *i*JO1366 model simulations. Seven mutations were observed in the intergenic region upstream of *galP* in five independent replicate experiments (Table 2). Of these seven mutation events, four were identical at the nucleotide level of mutation (Table 2), thus demonstrating a high degree of parallel evolution for these replicate experiments and implicating that these intergenic mutations played an important role in adaptation to the *ptsI* perturbation. The likelihood of getting the same mutation at the nucleotide level in two independent samples is even less likely than at the gene level (Fisher’s exact test *P* < 5e-06, Additional file 3).The mutation event was suggested to be associated with increased expression of *galP* via reduced repression by the transcriptional repressor GalR based on binding site analysis [31]. Other mutations observed across the replicate *ΔptsI* populations were in CRP (cyclic-AMP regulatory protein). Six unique *crp* mutations were observed in five replicate experiments (Table 2). CRP is known to regulate the transcription of approximately 100 genes, including *galP*, and it is activated by binding cyclic-AMP (cAMP) [32–34]. Thus, the mutations observed could be linked to influencing the expression of *galP*. Furthermore, RNA sequencing data showed that *galP* was up-regulated in *ΔptsI* strains as compared to wild type. The expression of *galP* was significantly up-regulated in the Exp. 1, Flask 5 clone assayed at an increase of 3.3X (log_2_(fold change) = 1.73), and further increased 1.9X (log_2_(fold change) = 0.908) in the Exp. 3, Flask 1 population analyzed. These increases in expression thus support the observation that *galP* does play a role in providing an alternate pathway for glucose transport in strains without a functional PTS system (Table 3). It is of interest to note, however, that a *cyaA* mutation was observed in the Keio parent strain used to inoculate all experiments and this is likely widespread in the Keio collection (Additional file 5). The mutation observed was a seven base-pair deletion leading to the truncation of the CyaA (cyclic-AMP synthase) protein, reducing it from 848 amino acids to 485 amino acids. CyaA activity is important for the activation of the regulator CRP [35, 36] and it is thus likely that this deletion event influenced these growth study results. A similar *cyaA* truncating mutation was observed in an adaptive laboratory evolution study focusing on the effects of removing *ptsH*, *ptsI*, and *crr* PTS genes [37]. It is also possible that the *cyaA* mutation could have resulted in a synthetic genetic interaction with those mutations acquired during the adaptation experiments.

The *ΔmetC* strains evaluated in the growth screen protocol showed mutations in only some of the populations sequenced (Table 2, no mutations were detected in Exp. 1). For *ΔmetC*, the model predicted that *malY* could compensate for the gene-deletion. The *ΔmetC* populations showed mutations in *malI*, a regulatory protein that represses expression of *malY* [38, 39]. Thus, it was hypothesized that the two mutations observed in independent replicate experiments are likely responsible for increasing *malY* expression.

Mutation analysis for the remaining cases did not show as clear of an agreement with model predictions. The mutations observed in the *ΔcarA* strains did not agree with the model-predicted alternate pathway; however, it did suggest agreement with previous multi-copy suppression results associated with over-expression of *carB* [19] (Table 1). One mutation observed in a *ΔcarA* population was in the *carA*/*carB* intergenic region, suggesting a regulatory effect on the expression of *carB*. Another was a synonymous mutation in the coding region of *carB*, indicating possible selective pressure for the use of *carB*, even though the protein sequence did not change. The synonymous mutation observed was for the Leucine 11 position, substituting the CUG codon with CUU. According to codon usage frequencies for *E. coli*, the CUG codon is in greater relative abundance (0.5) compared to that of the CUU codon (0.1) [40]. Thus, the mutation is not expected to improve translation efficiency based on codon frequency. Alternatively, such mutations could impact mRNA secondary structure and positively influence transcript levels, enzyme production, or enzyme activity as has been previously reported [41, 42]. Lastly, there was also a genome duplication event observed in some of the replicate experiments which included *carB*.

For the *ΔserB* experiments, distinct protein-coding, intergenic, and tRNA non-coding mutations were observed that could be linked to increasing the expression and possibly the activity of an isozyme, HisB. This isozyme relationship (i.e., *hisB* being an isozyme of *serB*) was not included in the *i*JO1366 reconstruction; alternatively, a pathway for L-serine biosynthesis linked to *glyA* was predicted by modeling to suppress the *serB* deletion (Table 1, Fig. 2c). Mutations observed in the replicate experiments, however, did not appear to be associated with the *glyA* alternate pathway (Table 2, Fig. 2c). Looking further, previous work has shown that plasmid over-expression of *hisB*, *gph*, and *ytjC* individually could rescue a *serB* KO strain [19]. Furthermore, directed evolution experiments have identified mutations in the corresponding enzymes (HisB, Gph, and YtjC) that could improve the isozyme activities that rescue a *serB* deletion [43]. One such mutation, a D57N HisB protein change [43], was also observed in a flask 5 clonal sample (i.e., a clone taken after several generations of growth of the starting strain) in this study (Fig. 2c, Additional file 4).

Parallel mutations linked to the regulation of the histidine operon were also observed in *ΔserB* flask 1 populations (Fig. 2c). Previous work has supported an attenuator model of regulation for the histidine operon [44–47]. Transcription of the histidine operon is believed to be dependent on the secondary structure of a lead mRNA (intergenic region between *hisL* and *hisG*), which is affected by the translation of a histidine-rich lead peptide (*hisL*). One key mutation observed was found in the *hisL*/*hisG* intergenic region of experiment 1 (Fig. 2c), likely increasing transcription of histidine operon genes (including HisB) by directly affecting the attenuator region. Indeed, differential gene expression analysis of RNA sequencing data for a *ΔserB* strain containing this mutation showed that *his* operon genes were significantly up-regulated compared to wild type expression (Fig. 3c). In particular, *hisB* expression showed a 13X increase in expression (log_2_(fold change) = 3.75) compared to wild type expression (Table 3). Four other replicate experiments, however, accrued four distinct mutations in *hisR*, a non-coding histidine tRNA (Table 2). Specifically, three (out of four) of these mutations were found in the acceptor-stem of tRNA ^*His*^ (Additional file 6)–a region of tRNA important for recognition by aminoacyl-tRNA synthetases (aaRS) [48, 49] and for proper cleavage of the pre-tRNA transcripts [50, 51]. Moreover, tRNA ^*His*^ position A71 interacts with multiple residues of Histidyl-tRNA synthetase (HisRS) [48] and the A–>G (72/77nt) mutation found in replicate 7 has been shown to decrease the cleavage precision of pre-tRNAs by *E. coli* ribonuclease P [50]. Previous studies have demonstrated that mature tRNA ^*His*^ can attenuate the transcription of the his operon genes [44–47]. Thus, the *hisR* mutations observed in the replicate experiments in this work are speculated to reduce the amount of mature tRNA ^*His*^ and its attenuator behavior upon the his operon by decreasing the efficacy of pre-tRNA ^*His*^ cleavage and amino-acylation, allowing for increased HisB expression. This proposed mechanism’s output of increased HisB expression was supported by examination of RNA sequencing data for a popultaion containing a *hisR* mutation (Fig. 3d). Differential gene expression analysis showed that the *his* operon genes were significantly up-regulated in the *ΔserB* population compared to wild type (Fig. 3d) and *hisB* showed a 9.5X increase in expression (log_2_(fold change) = 3.25) (Table 3). Overall, the highly reproducible mutations observed in this study appear to be linked to increasing expression and possibly the side-activity of HisB, a histidinol phosphatase which can also perform the phosphoserine phosphatase function of SerB [43].

### Structural mutations are indirectly linked to an underground activity

**Fig. 4:**
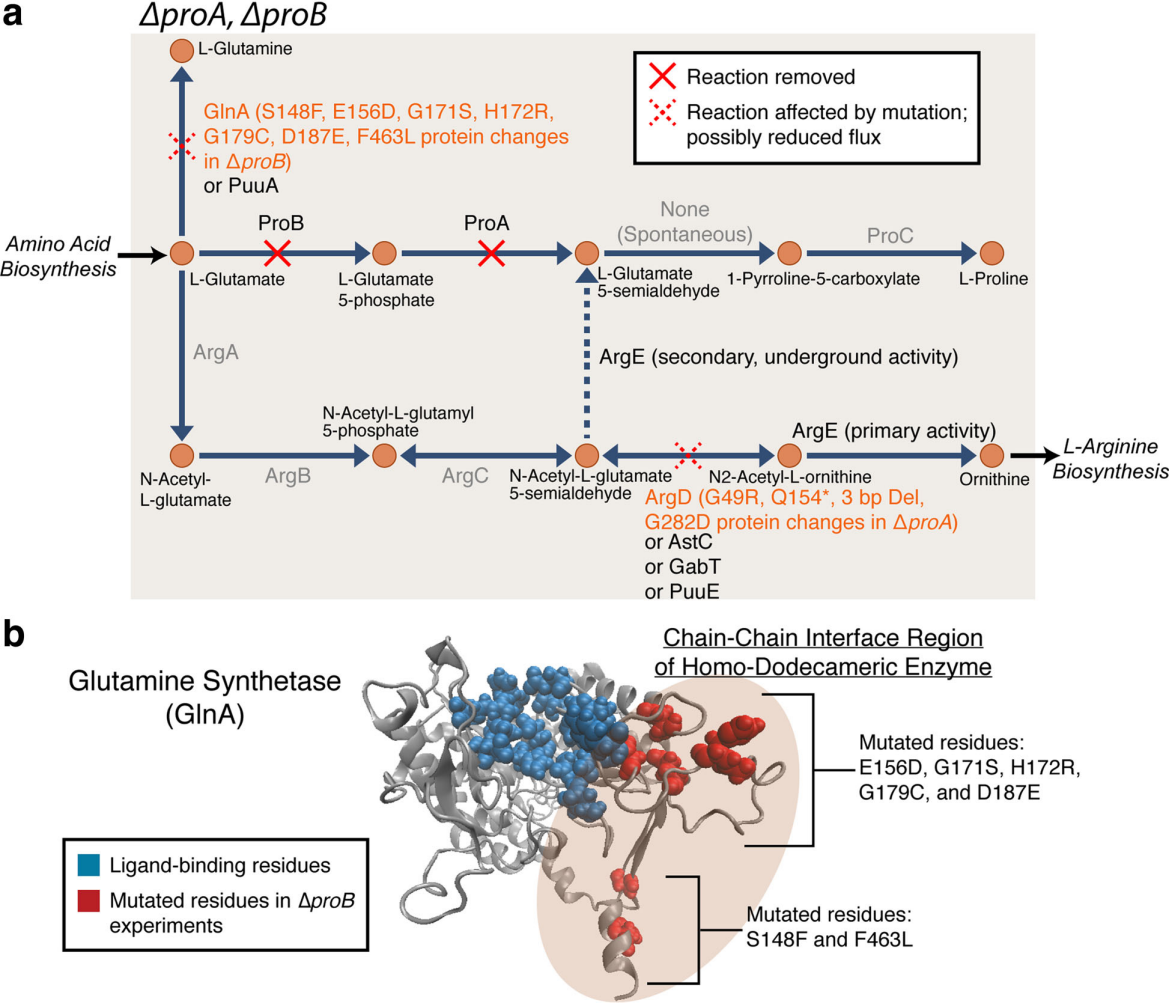
Structural mutations observed in *ΔproA* and *ΔproB* experiments analyzed in relation to ArgE underground activity. **a** Metabolic pathway maps related to *ΔproA* and *ΔproB* false positive cases. Both are involved in L-proline synthesis. Model simulations predict using an alternate pathway related to arginine and ornithine synthesis to rescue a *proA*/*proB* deficient *E. coli* strain. Mutations were observed in the coding regions of the metabolic genes *argD* and *glnA*. It is suggested that reduced flux through these enzymes increases flux through the ArgE associated underground activity, thus increasing production of L-proline and allowing for cell growth. **b** Mutation analysis in relation to the glutamine synthetase (GlnA) protein structure. An I-Tasser-predicted protein structure is provided [55] and the amino acid residue associated with observed *glnA* mutations in the *ΔproB* populations are highlighted in red. Those residues associated with ligand binding based on the crystal structure of the *Salmonella typhimurium* GlnA enzyme [56] are highlighted in blue. The mutations appear to be in buried regions of the homo-dodecameric enzyme at the interface of chain-chain interactions

The mutations observed in the *ΔproA* and *ΔproB* growth screen experiments could not be directly linked to the predicted alternate gene *argE* as in those FP cases previously discussed; however, analysis suggested that the mutations found in neighboring genes around the argE encoded reactions are indirectly related to the suppression of a *proA* or *proB* deletion phenotype. ProA and ProB are enzymes involved in the first two steps of proline biosynthesis in *E. coli* K-12 (Fig. 4a). Previous work in *Salmonella typhimurium* and *E. coli* strains [52–54] have suggested that an underground activity of the ArgE enzyme in *E. coli*, typically involved in the arginine biosynthesis pathway, can catalyze the conversion of N-acetyl-L-glutamate-5-semialdehyde to L-glutamate 5-semialdehyde (Fig. 4a) [20]. This side activity of ArgE does not typically occur at a significant enough level to rescue a *proA*/*proB* KO strain unless a mutation in *argD* occurs. The proposed mechanism of suppression is that a mutation inactivates ArgD activity leading to sufficient build up of the N-acetyl-L-glutamate-5-semialdehyde metabolite such that the underground activity of ArgE becomes significant [20, 54]. Thus, the four parallel mutation events in *argD* observed in the *ΔproA* replicate experiments are in agreement with these prior reports (Table 2). One observed mutation in this study was predicted to significantly affect ArgD activity by interfering with substrate binding since residues 283 and 284 have been identified as ligand binding residues and the mutation observed was of glycine 282 changing to aspartate. Other mutations observed in replicate experiments included a three base-pair deletion and introduction of an early stop codon (Fig. 4a), also likely to reduce ArgD activity.

While the mutations observed in the *ΔproA* experiments seemed to agree with previous reports, mutations observed in *ΔproB* appeared to be novel, but still related to the underground activity of ArgE. Eight mutations in seven independent *ΔproB* replicate experiments occured in the coding region of *glnA*, a glutamine synthetase encoding gene (Fig. 4a, Table 2). When the mutated amino acid residues are highlighted on a predicted GlnA protein structure (I-Tasser structure prediction [55]), they appear to be clustered in two distinct regions of the GlnA chain (Fig. 4b). The mutations do not appear to be directly changing ligand-binding residues based on analysis of corresponding ligand-binding residues of a *Salmonella typhimurium* GlnA enzyme [56]. The mutations appear to be in regions that are highly buried and involved in chain-chain interactions of the homo-dodecameric enzyme (Fig. 4b). These mutations are likely to have some effect on GlnA enzyme activity. If GlnA activity were reduced, it is suggested that a larger L-glutamate pool could increase flux through the ArgABCE pathway (Fig. 4a) and suppress the *proB* deletion. It is also of interest to note that there were apparent differences in growth fitness among three replicate growth curve experiments as seen in Fig. 1b and Additional file 2. The D187E mutation observed in Exp. 5 appeared to result in significantly greater fitness compared to the F463L mutations observed in Exps. 6 and 7 (Additional file 2, Table 2). These two mutations lie in distinct regions of the GlnA chain (Fig. 4b). In summary, mutation analysis for the *ΔproA* and *ΔproB* experiments suggested distinct adaptive mechanisms indirectly related to the low-level, underground activity of the ArgE enzyme. This alternate pathway is in agreement with model predictions made with *i*JO1366.

### Genome amplification events

A mechanism of adaptation observed in several gene KO growth experiments was genome amplification. This type of adaptation was observed most clearly in some of the *ΔcysK*, *ΔptsI*, and *ΔcarA* replicate experiments. Examination of the functional significance of these large duplication events is statistically less compelling compared to small mutation events (Fisher’s exact test resulting in larger *p*-values depending on size of duplication, Additional file 3). However, although these large regions of amplification often contain hundreds of genes, their impact can sometimes be linked to a key gene of interest [14, 57, 58]. The largest duplication observed was in a *ΔcysK* flask 1 population (Table 2). CysK is a PLP-dependent enzyme involved in L-cysteine biosynthesis and has been annotated as a cysteine synthase and L-cysteine desulfhydrase [59, 60]. There are multiple cysteine-desulfhydrases suspected for *E. coli* K-12 including *cysM*, *metC*, *tnaA*, and *malY* [61]. CysM was the isozyme listed in *i*JO1366 and predicted to rescue a *cysK* KO strain. Mutations in the flask 1 population sample for the *cysK* KO strain did not reveal any clear key small mutations; however, a large region of genome amplification of 2X multiplicity was observed based on read-depth analysis (Additional file 7). The region spanning approximately 2 million basepairs, or slightly less than half of the genome, does contain the gene encoding the model-predicted alternate isozyme, *cysM*. The region of amplification was flanked by IS186 insertion elements (Additional file 7). It is proposed that these repetitive IS element sequences were instrumental in the mechanism of duplication by recombination as has been previously described [57]. Although *cysM* was included within the large region of amplification, further follow-up studies conducting expression and/or KO analysis are required in order to make more definitive claims of the alternate pathway used to compensate for the *cysK* gene deletion.

Two instances of genome duplication occurred in replicate experiments of *ΔcarA* and *ΔptsI* (Additional file 7b, c). For the *ΔcarA* populations, a region of approximately 520 kilo base pairs was duplicated in two replicate experiments (Table 2). This region of amplification was flanked by IS2 insertion elements. The region that was amplified does include the *carB* gene that has been shown to suppress a *carA* gene deletion in previous work [19] (Fig. 4b). Thus, similar to the *cysK* case, the repetitive IS2 element seems to mediate the amplification and increased dosage of the enzyme encoded by *carB* (Fig. 4b). Unlike the *cysK* and *carA* genome amplification events, the *ptsI* amplification was significantly smaller (99 kilo base pairs) and flanked by genes encoding ribosomal RNA (*rrlC* and *rrsC* on one side and *rrlA* and *rrsA* on the other) (Fig. 4c). The gene pairs *rrlA* and *rrlC*, and *rrsA* and *rrsC* each share 99% sequence identity according to BLAST (basic local alignment search tool) alignment analysis [62]. Thus, these repetitive sequence regions are potential targets for duplication by recombination as is observed with IS elements [57]. Although this region did not contain the model-predicted gene of interest, *galP*, it did contain the gene *cyaA*, which encodes an adenylate cyclase. Adenylate cyclase is responsible for the synthesis of cyclic AMP, which is an important signaling molecule, and as previously mentioned, important for activation of the regulator CRP [34–36]. Thus, this amplification appeared to be indirectly related with affecting expression/regulation of *galP*.

### FP strains requiring no mutations for growth, or, true positives

For those strains that did not acquire detectable mutations during the long growth experiments, *ΔubiE* and *ΔcysP*, it is assumed that only regulatory responses were required to shift expression of alternate metabolic pathways and enable growth. For the case of *ΔubiE*, however, the drastic reduction in final cell density observed in replicate experiments suggests that the associated reactions involved in ubiquinone and menaquinone biosynthesis are important for cellular energetics [63]. Previous work has shown that *ubiE* mutant strains can grow using demethylmenaquinone as the sole respiratory quinone [64]. Although reported to be important during anaerobic growth, demethylmenaquinone was observed to have a small but significant capacity to function during aerobic growth as well [65]. Furthermore, previous high throughput growth screens show inconsistencies in labeling *ΔubiE* as essential, probably due to cell density cut-offs utilized to label growth/ no growth [7, 12, 66]. Overall, the results of this study (Fig. 1b, Table 1) are consistent with prior reports and show that *ubiE* is non-essential for growth on glucose minimal medium. For the case of *cysP*, it is possible that the predicted alternate pathway (Table 1) could be used to enable growth and only regulatory shifts already wired in the wild-type strain are required. Detailed analysis of these regulatory shifts were not pursued in this study; however, future work could examine expression (RT-qPCR, RNAseq) of model-predicted alternate pathways, following workflows similar to those previously reported [14]. These cases are no longer considered FP model predictions, but instead were true positive predictions in agreement with the model.

## Discussion

This study utilized a systematic model-driven approach to identify genes that were mistakenly labeled as essential in minimal media, as well as interpret and suggest mechanisms of adaptation to such genetic perturbations when combined with growth experiments, whole genome, and transcriptome sequencing. Three key findings were supported by the results. Firstly, extended growth tests of gene KO strains were shown to result in the reversal of several calls of essentiality, in agreement with model predictions. This finding has direct implications to high-throughput screens of essentiality. Secondly, it was demonstrated that mutation events are likely even after relatively short incubation times in response to genetic perturbations. Finally, results showed that analysis of parallel mutation events among replicate experiments have implications for expanding gene-protein-reaction associations in both knowledge bases and models.

Growth/ no growth calls made by large-scale growth screens of gene-deletion strain collections such as the Keio collection [7] serve as a comprehensive guide for strain are key to testing the predictive power of genome-scale metabolic models [13, 67]. Cellular acclimation to such genetic and metabolic disruptions may require greater time to make such growth/no growth calls as mechanisms of adaptation or regulatory responses might be required for detectable growth. Extended growth incubation of FP KO strains in this study revealed that 55% (11 out of 20) of the FP strains available and confirmed could be considered true positives (both experiments and predictions in agreement with calls of non-essentiality). Growth of the examined FP KO strains was reproducible, the degree of which is outlined in Table 1, given growth conditions that were well-aerated and provided sufficient time to allow for extended lag-phases. Thus, this study outlines a quantitative time window in which high-throughput growth screens can be designed to call growth/no growth phenotypes going forward [7, 66, 68]. There was a great deal of phenotypic diversity observed for the different KO strains that grew sub-optimally (as compared to wild type) and this diversity is manifested in the different mechanistic responses of the cells as revealed through mutations. This wide range in growth fitness demonstrates that essentiality screens for binary growth/no growth do not provide a complete view of a more general contribution to fitness. The idea of applying a ‘fitness index’ to each gene has been demonstrated previously [69] and may allow for a more quantifiable metric of contribution to growth fitness. Furthermore, the nine strains which were confirmed as FPs and were conditionally essential under these conditions are either likely truly unable to grow under the conditions, or could require more complex adaptation (i.e., mutations) to activate alternative pathways. Such efforts could be explored using many more replicates, engineered hypermutating strains, or by introducing a significant number of mutations to a given strain prior to a growth test with a method such as UV mutagenesis [70]. Alternatively, the model could indeed contain ‘non-physiological’ reactions and this set of nine cases provides a focused set of strains to explore such events, in detail [71]. Lastly, the three genes not in the Keio collection set or confirmed using the outlined PCR validation, *aroE*, *entD*, and *waaU*, which were also not deemed essential on rich media [22] could be attempted to be constructed and put through the same workflow established in this work.

Coupling population sequencing with extended growth tests in this study revealed that mutation events of interest were likely, even within a period of incubation as short as 48 h. The FP strains that were considered for this study were ultimately placed in one of three categories of essentiality: conditionally essential, non-essential, and non-essential with mutations. Those strains that were repeatedly able to grow given longer periods of incubation were considered non-essential (with or without mutations) and thus in agreement with model predictions of growth (i.e., reassigned as true positives). Of these strains, mechanisms of adaptation to genetic perturbation were categorized broadly as either requiring mutations or not requiring mutations for growth. Population sequencing and mutation analysis of the FP KO strains revealed that 82% (9 out of 11) of the strains that grew accrued mutations in at least some of the replicate population samples sequenced (Fig. 1). This result is of general interest as short-term growth screens are commonly practiced with the assumption that mutations are not acquired during such short periods of growth. A set of KO strains which accrued mutations could be linked directly or indirectly to predicted suppression phenotypes (see below). For those populations that did not accrue mutations, it is suggested that the annotated alternate pathways or isozymes listed in the genome-scale reconstruction and model of metabolism utilized in this study were likely correct. However, such confirmation of unmutated KO strains was not the focus of this study and future work could examine this more comprehensively by performing similar additional cellular measures such as expression analysis of the predicted isozymes, as was shown for a subset of cases here and has been previously demonstrated [14], or a complementary approach such as ribosomal sequencing [72]. Furthermore, it is also of general interest to note that some starting Keio strains grown and isolated on a nutrient rich medium possessed mutations that may have influenced growth on the minimal medium(Additional file 5). There is strong evidence for this in the *ptsI* KO strain in the Keio collection. Given the wide usage of such gene-deletion libraries, it is important to understand baseline mutations and how they may influence downstream applications.

Examination of mutational parallelism at the gene level proved to be informative and provided compelling contextual evidence for correlation to modeling predictions in a number of the gene KO cases examined. For those strains that did require mutations, the mutations observed across replicate experiments (Table 2) allowed for the identification of proposed alternate pathways. Six of the nine cases examined (*ΔthrA*, *ΔptsI*, *ΔproA*, *ΔproB*, *ΔmetC*, and *ΔcysK*) showed key mutations that were interpreted to be in agreement with the model-predicted alternate pathways, thus allowing us to label them as newly assigned true positives. Mutation enrichment across replicate experiments has been shown to provide strong evidence that they were positively selected for [23, 24], and this coupled with previously reported data provided the basis for the proposed mechanisms of adaptation described in this study. The establishment of causality for each gene KO strain in detail, however, will require follow-up experiments isolating individual mutants and conducting more detailed experimental analysis as has been previously demonstrated [73]. The results and mutations identified here are the starting point for such studies. Furthermore, there are additional key mutations which were identified to display parallel evolution (e.g., *metK* in the *ΔmetL* strain) whose mechanism of adaptation was not immediately obvious and such cases provide additional targets for discovery (see Table 2).

## Conclusions

Adaptive flexibility is critical for organisms evolving to novel ecological niches or responding to environmental stress. When examining gene essentiality for applications such as drug discovery or modifying industrial bioprocessing strains, one must consider possible unanticipated adaptive mechanisms that may follow the intended genetic disruption. Underlying enzymatic side activities may rise to the surface after short adaptive periods leading to unwanted ‘rogue’ activities [74]. This study shows that while high-throughput, short-term growth screens may capture a large-scale picture of gene essentiality, they may not reveal underlying metabolic capabilities attainable with slightly longer incubation or short adaptive periods. Furthermore, these findings suggest that many of the strains in large gene-deletion collections, such as the Keio collection, likely contain adaptive mechanisms to overcome the intended KOs. Thus, sequencing is likely necessary prior to using such clones for the myriad applications they enable. In conclusion, the results presented in this study highlight genetic and metabolic flexibility in response to gene disruption in the organism *E. coli*. Furthermore, genome-scale reconstructions and metabolic models provide a promising avenue for the elucidation of adaptive mechanisms and for predicting observable in vivo phenotypes.

## Methods

### False positives selection and *In silico* model validation

The false positive strains identified for longer growth tests were taken from the previously published work [13]. The strains listed in Additional file 1 are the subset of genes considered in this study. They were described as false positive predictions on at least one substrate examined and had no experimental growth on any of 34 substrates experimentally tested [13]. However, upon further examination, it was observed that *ΔcysK* and *ΔcysP* did have experimental evidence of growth on glucose carbon source [7, 66]. These two cases were thus examined using a glycerol substrate on which they were still considered false positive predictions [68].

The false positive predictions were verified as growth predictions *in silico* by utilizing the comprehensive metabolic reconstruction of *E. coli* K-12 MG1655, *i*JO1366 [10]. The flux balance analysis (FBA) simulations were conducted using the constraint-based modeling package COBRApy [75]. Simulations were conducted by optimizing the core biomass objective function, which is determined to be a stoichiometric representation of all core metabolic biomass components in the cell [66]. In order to better match the genotype of the BW25113 Keio Collection parent strain adjustments were made to the *i*JO1366 model to reflect missing genes (*araBAD*, *rhaBAD*, and *lacZ*). Flux through reactions ARAI, RBK _L1, RMPA, LYX1, RMI, RMK, and LACZ was constrained by setting reaction bounds to zero as has been previously performed [10, 12]. To simulate a gene-deletion growth screen and thereby closely mimic experimental growth conditions, the desired gene was removed from the metabolic model and then a FBA simulation was run as previously described [13], setting the glucose (or glycerol) exchange reaction lower bound to -10 mmol · gDW ^−1^*h*^−1^ (gDW is an abbreviation of gram of dry weight) and the oxygen exchange reaction lower bound to -1000 mmol · gDW ^−1^*h*^−1^. All gene-deletion simulations were verified to result in a prediction of growth in agreement with previous reports [13]. All genes listed in Additional file 1 were confirmed to be false positives and FBA simulation results of isozymes and alternate pathways are additionally provided for a subset of genes from Table 1 in Additional file 8.

Isozymes and alternate pathways listed in Table 1 were determined based on analysis of *i*JO1366. Isozymes were extracted from the gene-protein-reaction (GPR) associations for each reaction in the model, where a Boolean “OR" relationship indicates a set of isozymes. Alternate pathways were identified by knocking out a target pathway responsible for production of an essential biomass component, running FBA with this pathway knocked out, and looking for alternative reaction flux that enables production of the essential biomass component. These alternative pathways are also present in the EcoCyc knowledge base [76] with the exception of *serB* (Fig. 2c). These isozymes and alternate pathways are also listed and described in a previous publication [13].

### Strains utilized and PCR verification

All strains utilized in this study were taken from the single-gene deletion Keio collection [7]. The strains examined in this work are listed in Additional file 1. These strains are all derived from the parent Keio strain *E. coli* K-12 BW25113. The reference strain utilized in growth screens and wherever ‘wild type’ is specified in this manuscript is the parent Keio strain without any deletions or Kanamycin resistance cassette.

The strains utilized in the growth screens were first verified by polymerase chain reaction (PCR) experiments utilizing the methods detailed in [7]. For each strain that was used, they were verified by three PCR reactions utilizing 1)flanking primers, 2) internal K1 and forward flanking primer, and 3) internal K2 and reverse flanking primer as previously suggested in [7].

### Culture conditions and growth characterizations

Rich media utilized for pre-culture growth was Luria-Bertani Broth (LB). LB media consisted of an autoclaved 25 g/L LB Broth (EMD Millipore LB Broth, Miller - Novagen, catalog 71753) in Milli-Q water. The M9 minimal media utilized in the long term growth characterizations consisted of 0.1mM CaCl_2_, 2mM MgSO_4_, 1x Trace elements Solution, 1x M9 salts solution, and either 2g/L glucose or 0.2% (by volume glycerol), in Milli-Q water. The 4000x trace elements solution consisted of 27 g/L FeCl_3_ · 6H_2_O, 1.3 g/L ZnCl_2_, 2 g/L CoCl_2_ · 6H_2_O, 2 g/L Na_2_MoO_4_*2H_2_O, 0.75 g/L CaCl_2_, 0.91 g/L CuCl_2_ · 2H_2_O, and 0.5 g/L H_3_BO_3_, in concentrated HCl. The 10x M9 salt solution was composed of 68 g/L Na_2_HPO_2_, 30 g/L KH_2_PO_2_, 5 g/L NaCl, and 10 g/L NH4Cl, in Milli-Q water. The M9 media, trace elements solution, and M9 salt solutions were all sterile filtered. Except for the BW25113 wild-type strain, all LB and M9 cultures contained 25 mg/L Kanamycin A.

The twenty strains that were available in the Keio collection and PCR verified (Additional file 1), were selected for an initial long-term growth test. Pre-cultures of these strains were grown overnight in 2-3 mLs of LB media in a 10 mL culture tube on a shaker plate. The following morning, 50 mL M9 minimal media cultures in 250-mL Erlenmeyer flasks containing magnetic stir bars for aeration were inoculated at a target OD600 of 0.01-0.02. The OD600 was monitored at least once a day for two weeks or until growth was observed, at which point the cells were passed to a new flask of M9 minimal media to ensure that the growth observed persisted. The cells were passed consecutively to 5 flasks to ensure the growth observed was consistent. At the end of the experiment, glycerol stock samples were frozen at -80 ^∘^C for future use and the flask 5 population was PCR validated as described above to ensure there was no contamination.

Following the initial growth screen, a more detailed growth characterization was conducted on an automated platform. Initial 15 mL LB pre-cultures were inoculated from glycerol frozen stocks of the Keio KO Collection strains and the Keio KO parent strain BW25113, and were grown overnight. Growth test cultures were then started in triplicate by pipetting 50 *μ*L from a preculture into three 17 mL tubes containing M9 minimal media. Both the pre-cultures and growth tests were grown at 37 ^∘^C in magnetically stirred tubes, at a rate of 1,100 rpm to ensure full aeration. Optical density at 600 nm (OD600) sampling was performed using an automated system with a Tecan Sunrise Microplate Reader, using 100 *μ*L of culture for each measurement. Sampling frequency was initially between 6-12 h, and was increased to 2-4 h once growth was observed. The OD600 data was then converted to units of grams of dry weight per liter (gDW/L) using the conversion factor for the plate reader and sample volume (1.663 gDW/L/OD600). The growth curves depicted in Fig. 1b were constructed by importing cell density data from the experiment into a Jupyter notebook (http://jupyter.org/) and utilizing the scientific computing library suite SciPy (http://www.scipy.org/).

### Whole genome sequencing and mutation analysis

Genomic DNA was isolated using a Macherey-Nagel NucleoSpin Tissue Kit. DNA concentrations were determined using a Thermo Fischer Qubit dsDNA HS Assay Kit. Paired-end whole genome DNA sequencing libraries were prepared using a Kapa Biosystems KAPA HyperPlus Kit. Manufacturer protocols were followed for all kits. DNA sequencing libraries were then run on a Illumina HiSeq4000 platform with a 100/100 HiSeq 3000/4000 PE cycle kit (PE-410-1001).

The *breseq* pipeline [77] version 0.30.0 with bowtie2 version 2.2.6 [78] was used to map sequencing reads and identify mutations relative to the *E. coli* BW25113 genome (NCBI accession CP009273.1). For the samples examined in this study, the average of percent mapped reads was 95% and average mean coverage was 130 reads. The pre-existing mutations observed in the knockout strains prior to growth characterization experiments (compared to the wild type BW25113 reference strain) are provided in Additional file 5. Mutations considered for analysis in this study were present in a population at a fraction > 0.2. Those mutations listed in Table 2, were further filtered so the key mutations discussed in the results are presented. The complete list of mutations observed above the population fraction cut-off is provided in Additional file 4. Additionally, analysis of large regions of genome duplication was performed by analyzing read depth coverage utilizing a custom python script. Contingency table analysis shown in Additional file 3 running Fisher’s exact tests were conducted using conducted using the scientific computing Python library SciPy (http://www.scipy.org/) in a Jupyter Notebook (http://jupyter.org/).

### RNA sequencing

RNA sequencing data were generated under aerobic, exponential growth conditions on M9 minimal medium plus 2 g/L D-glucose and 50 mg/L Kanamycin A. The wild type BW25113 strain control was grown in the absence of antibiotics. Culture conditions and growth conditions were the same as those used in the automated characterizations described above. Pre-cultures for the RNA sequencing experiments were taken by scraping frozen stocks (taken from the growth characterization experiments) and growing all strains in M9 minimal medium plus 2 g/L D-glucose. The pre-cultures were split into two 17 mL tubes (biological duplicates) for each experimental condition. Cells were harvested in exponential growth using the Qiagen RNA-protect bacteria reagent according to the manufacturer’s specifications. Pelleted cells were stored at -80 C prior to RNA extraction. Cell pellets were thawed and incubated with lysozyme, SuperaseIN, protease K, and 20% sodium dodecyl sulfate for 20 min at 37 C. Total RNA isolation and purification was performed using Qiagen’s RNeasy minikit column according to the manufacturer’s specifications. After total RNA isolation, its quality was checked with the Agilent Bioanalyzer using their RNA 6000 nano kit. Ribosomal RNA (rRNA) was removed using Epicentre’s Ribo-Zero rRNA removal kit for Gram-negative bacteria. Paired-end, strand-specific RNA sequencing libraries were generated using Kapa Biosystem’s KAPA RNA HyperPrep Kit. The final pool of libraries was subjected to a 1x SPRI (Solid Phase Reversible Immobilization) bead size selection to further remove unincorporated PCR primers. RNA sequencing libraries were run on an Illumina NextSeq 550 machine using a NextSeq 500/550 Mid Output v2 kit (150 cycles).

For each condition examined, biological duplicate samples were sequenced and analyzed. RNA sequencing reads were mapped to the *E. coli* BW25113 genome (CP009273.1) using bowtie2 version 2.3.4.1 [78] and the following options: bowtie2 -X 1000 -N 1 -p 20 -3 3 -1 <R1 files> -2 <R2 files> -x < index>. The SAM output file of bowtie2 was converted to BAM and sorted using samtools version 1.8 [79]. The average total number of reads per sample was 9.8 million reads, with the minimum number of reads for one sample being 6.8 million reads and the maximum number of reads for one sample being 17 million reads. All samples had an alignment rate greater than 97.5%. The read counts per gene for each sample were calculated using the summarizeOverlaps function from the R package GenomicAlignments in Bioconductor. The input options for the summarizeOverlaps function were as follows: mode=“Union”, singleEnd=FALSE, ignore.strand=FALSE, fragments=FALSE. The raw counts calculated by the summarizeOverlaps function were used to identify differentially expressed genes using the DESeq and results functions from the R package DESeq2 in Bioconductor. The results function extracted log_2_(fold change) data and p-adjusted values from the DESeq analysis allowing for comparison of the experimental condition expression data (biological duplicate replicates) to wild type BW25113 expression data (biological duplicate replicates). The p-adjusted values used to construct Fig. 3 and displayed in Table 3 accounted for multiple testing correction using the Benjamini and Hochberg method, which is the default “BH" option for the DESeq2 results function. The independent filtering option for the results function was set to FALSE to obtain p-adjusted values for all genes. Gene expression fold changes were considered significant if the log_2_(fold change) was greater than 1 and the calculated p-adjusted value was smaller that 0.05. Volcano plots were constructed for the *serB* and *thrA* experiments (Fig. 3) in a Jupyter Notebook using (http://jupyter.org/) and the Python plotting library Matplotlib [80]. The RNAseq data is available in the Gene Expression Omnibus (GEO) database under the accession number GSE117303.

## Additional files

Additional file 1 All FP KO strains considered in this study. This file (.pdf) contains a table listing all false positive KO strains considered for extended growth tests. Additional information regarding proposed conditional essentiality is also provided as well as a comparison to a previous study [22]. (PDF 67 kb)

Additional file 2 Growth curves of five false positive replicate strains (*ptsI*, *serB*, *proA*, *proB*, and *carA*) in separate subplots are displayed. This file (.pdf) contains a figure that is an extension of Figure 1B, as the same curves for these cases are displayed; however, specific labeling of each curve with their associated experiment (exp.) number is provided in the legend of each subplot. The experiment number corresponds to those numbers listed in Table 2. (PDF 183 kb)

Additional file 3 Contingency table examples showing the likelihood of mutational convergence. This file (.pdf) contains a figure of contingency table examples showing the likelihood of mutational convergence between two samples. *p*-values calculated from Fisher’s exact test are reported for four examples. (PDF 97 kb)

Additional file 4 Mutations Summary for each FP case. This file (.xlsx) contains all mutations found for each false positive case described in this study. (XLSX 52 kb)

Additional file 5 Mutations observed in starting strains grown in LB rich medium. This file (.pdf) contains a table listing mutations identified in the PCR-confirmed Keio strains that were used in the extended growth tests on minimal medium. (PDF 49 kb)

Additional file 6 Secondary structure of tRNA ^*His*^ and observed hisR mutations. This file (.pdf) contains a figure showing the secondary structure of tRNA ^*His*^ and observed hisR mutations aligned with tRNA numbering convention. (PDF 272 kb)

Additional file 7 Genome duplication amplification events observed in *cysK*, *carA*, and *ptsI* experiments. This file (.pdf) contains a figure showing the read depth coverage (y-axis) plotted against the genome position (x-axis) for flask 1 population samples for A. *cysK*, B. *carA*, C. and *ptsI* experiments. (PDF 316 kb)

Additional file 8 FBA simulations for genes listed in Table 1. This file (.pdf) contains a table of simulations demonstrating the same predicted behavior in both the *i*JO1366 model corresponding to the MG1655 genotype and a modified *i*JO1366 model corresponding to the BW25113 genotype.(PDF 79 kb)

## Abbreviations

cAMP: Cyclic-AMP
CRP: Cyclic-AMP regulatory protein
FBA: Flux balance analysis
FP: False positive
GDA: Genome duplication amplification
gDW: Gram of dry weight
GPR: Gene-protein-reaction
KO: Knockout
LB: Luria-Bertani Broth
OD600: Optical density at 600 nm
PCR: Polymerase chain reaction
St. Dev.: Standard deviations
TPM: Transcripts per million
WT: Wild type
%RSD: Percent relative standard deviations
