# Supplementary material for: Reframing gene essentiality in terms of adaptive flexibility
Source: BMC Syst Biol. 2018 Dec 17;12:143. doi: 10.1186/s12918-018-0653-z (PMC6296033; doi:10.1186/s12918-018-0653-z)
Supplement: Supplementary file 1 — All FP KO strains considered in this study. This file (.pdf) contains a table listing all false positive KO strains considered for extended growth tests. Additional information regarding proposed conditional essentiality is also provided as well as a comparison to a previous study [22]. (PDF 67 kb) [file 12918_2018_653_MOESM1_ESM.pdf]

Additional File 1: Table of all FP KO strains considered in this study

Gene / Keio Strain	In Keio Collection/ Confirmed by PCR? Yes (Y) or No (N)	Growth? Yes (Y) or No (N)	Proposed Conditional Essentiality Yes (Y) or No (N)	Essential based on Goodall et al. 2018 on LB Yes (Y) or No (N) or Unclear (U)
aroE	Y/N	-	-	N
carA	Y/Y	Y	N	N
carB	Y/Y	N	Y	N
cysA	Y/Y	N	Y	N
cysK*	Y/Y	Y*	N	N
cysP*	Y/Y	Y*	N	N
glnA	Y/Y	N	Y	N
glyA	Y/N	-	-	Y
guaB	Y/Y	N	Y	U
ilvA	Y/Y	N	Y	N
metC	Y/Y	Y	N	N
metE	Y/Y	N	Y	N
metL	Y/Y	Y	N	N
pdxH	Y/Y	N	Y	Y
proA	Y/Y	Y	N	N
proB	Y/Y	Y	N	N
ptsI	Y/Y	Y	N	Y
pyrD	Y/Y	N	Y	N
serA	Y/Y	N	Y	N
serB	Y/Y	Y	N	N
thrA	Y/Y	Y	N	N
ubiE	Y/Y	Y	N	Y
folA	N/-	-	-	Y
can	N/-	-	-	Y
pyrH	N/-	-	-	Y
lnt	N/-	-	-	Y
fldA	N/-	-	-	Y
fabA	N/-	-	-	Y
acpS	N/-	-	-	Y
ppa	N/-	-	-	Y
folD	N/-	-	-	Y
entD	N/-	-	-	N
pgsA	N/-	-	-	Y
nrdA	N/-	-	-	Y
nrdB	N/-	-	-	Y
waaU	N/-	-	-	N
wzyE	N/-	-	-	Y
ubiB	N/-	-	-	Y

*cysK and cysP strains were grown using glycerol as the carbon source. Although they were listed as FP on all substrates considered in (Orth and Palsson 2012), they had previously been shown to grow on glucose, but not glycerol.
