# Supplementary material for: Reframing gene essentiality in terms of adaptive flexibility
Source: BMC Syst Biol. 2018 Dec 17;12:143. doi: 10.1186/s12918-018-0653-z (PMC6296033; doi:10.1186/s12918-018-0653-z)
Supplement: Supplementary file 2 — Growth curves of five false positive replicate strains (ptsI, serB, proA, proB, and carA) in separate subplots are displayed. This file (.pdf) contains a figure that is an extension of Figure 1B, as the same curves for these cases are displayed; however, specific labeling of each curve with their associated experiment (exp.) number is provided in the legend of each subplot. The experiment number corresponds to those numbers listed in Table 2. (PDF 183 kb) [file 12918_2018_653_MOESM2_ESM.pdf]

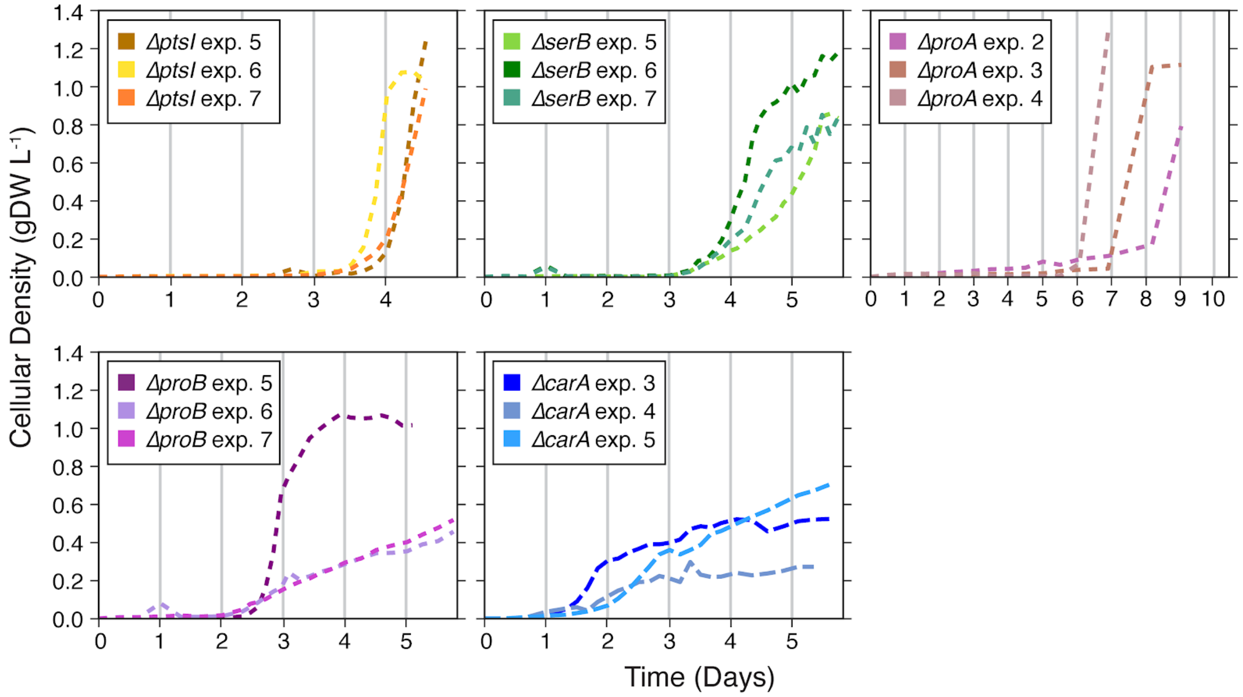

Additional File 2: Growth curves of five false positive replicate strains (ptsI, serB, proA, proB, and carA) in separate subplots are displayed. This figure is an extension of Figure 1B, as the same curves for these cases are displayed; however, specific labeling of each curve with their associated experiment (exp.) number is provided in the legend of each subplot. The experiment number corresponds to those numbers listed in Table 2. These five cases were chosen because of the growth fitness variability observed between replicate experiments. Growth data is displayed in terms of cellular density in grams of dry weight per Liter (gDW/L). Those strains that accrued mutations in all replicate populations during this growth test are noted with small dashed lines. The carA experiments showed mixed results, showing mutations in only some populations, is noted with larger dashed lines. All Keio strains were grown in M9 minimal medium with glucose as the carbon source.
