# Supplementary material for: Reframing gene essentiality in terms of adaptive flexibility
Source: BMC Syst Biol. 2018 Dec 17;12:143. doi: 10.1186/s12918-018-0653-z (PMC6296033; doi:10.1186/s12918-018-0653-z)
Supplement: Supplementary file 3 — Contingency table examples showing the likelihood of mutational convergence. This file (.pdf) contains a figure of contingency table examples showing the likelihood of mutational convergence between two samples. p-values calculated from Fisher’s exact test are reported for four examples. (PDF 97 kb) [file 12918_2018_653_MOESM3_ESM.pdf]

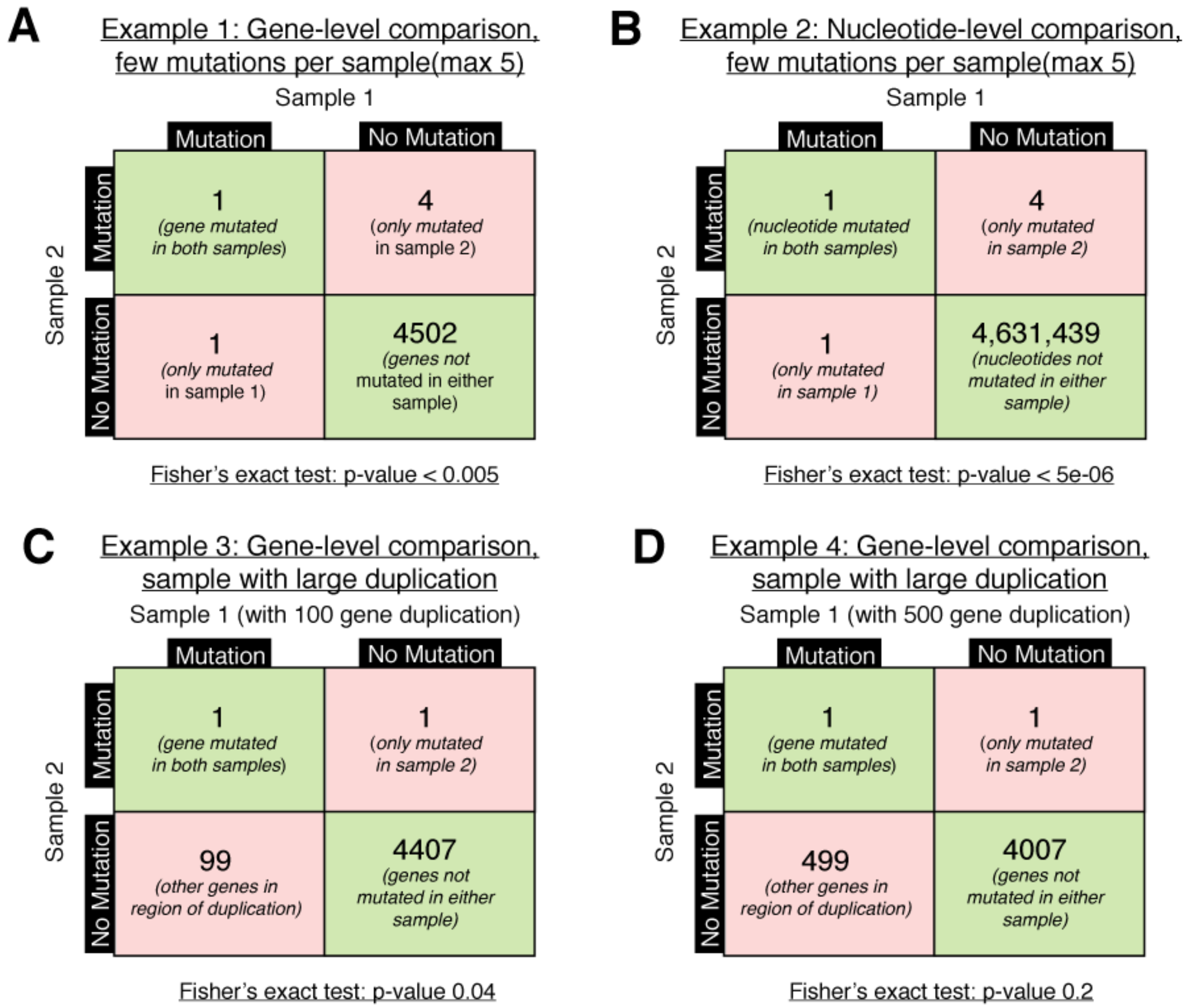

Additional File 3: Contingency table examples showing the likelihood of mutational convergence between two samples. A) Fisher's exact test P-value was calculated for the example of two samples acquiring a single mutation in the same gene. This was a proof of principle exercise for showing the significance of using parallel evolution events as a driver for mutation analysis. The greatest number of mutations observed in a single sample was 5 in the ptsI experiment. For other cases with fewer mutations, the P-value would be smaller. B) Fisher's exact test P-value was calculated for the example contingency table of two samples acquiring identical mutations at the nucleotide-level. Such an event was observed in the ptsI experiments. C) Fisher's exact test P-value was calculated for the example case of observing a large duplication event containing 100 genes in one sample. D) Fisher's exact test P-value was calculated for the example case of observing a large duplication event containing 500 genes in one sample. C) and D) show that the significance of the shared mutation even between two samples varies greatly depending on the size of the duplication event.
