# Supplementary material for: Reframing gene essentiality in terms of adaptive flexibility
Source: BMC Syst Biol. 2018 Dec 17;12:143. doi: 10.1186/s12918-018-0653-z (PMC6296033; doi:10.1186/s12918-018-0653-z)
Supplement: Supplementary file 5 — Mutations observed in starting strains grown in LB rich medium. This file (.pdf) contains a table listing mutations identified in the PCR-confirmed Keio strains that were used in the extended growth tests on minimal medium. (PDF 49 kb) [file 12918_2018_653_MOESM5_ESM.pdf]

Additional File 5: Mutations observed in starting strains grown in LB rich medium

**Gene / Keio Strain**	**Mutation(s)**	**Gene Mutated**	**Annotation**
carA	IS2 (+) +5 bp	lit	Coding (769-773 / 894 nt)
cysK	9 bp deletion	lrhA	Coding (85-93/939 nt)
metC	+C	yifE	Coding (150/339)
ptsI	A->G	trpL/yciV	Intergenic (-54/-84)
	(ATCAGCC)2->1	cyaA	Coding (1436-1442/2547 nt)
