# Supplementary material for: Reframing gene essentiality in terms of adaptive flexibility
Source: BMC Syst Biol. 2018 Dec 17;12:143. doi: 10.1186/s12918-018-0653-z (PMC6296033; doi:10.1186/s12918-018-0653-z)
Supplement: Supplementary file 6 — Secondary structure of tRNA His and observed hisR mutations. This file (.pdf) contains a figure showing the secondary structure of tRNA His and observed hisR mutations aligned with tRNA numbering convention. (PDF 272 kb) [file 12918_2018_653_MOESM6_ESM.pdf]

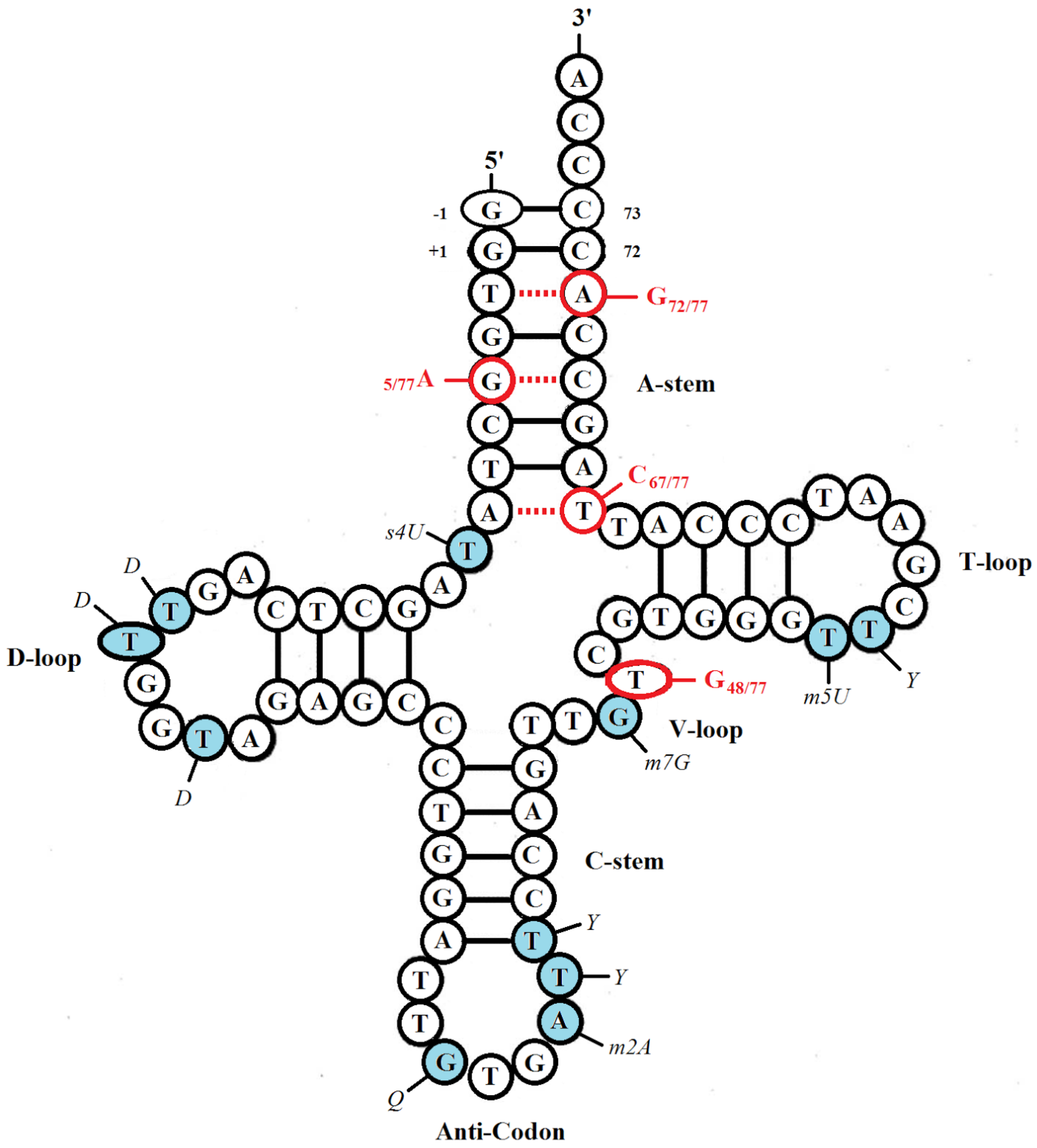

Additional File 6: Secondary structure of $tRNA^{His}$ and observed hisR mutations aligned with tRNA numbering convention (black numbering). tRNA modifications (blue) and observed mutations (red) are highlighted. Instability of structure caused by hisR mutations are in the acceptor stem (red dotted line).
