# Supplementary material for: Reframing gene essentiality in terms of adaptive flexibility
Source: BMC Syst Biol. 2018 Dec 17;12:143. doi: 10.1186/s12918-018-0653-z (PMC6296033; doi:10.1186/s12918-018-0653-z)
Supplement: Supplementary file 7 — Genome duplication amplification events observed in cysK, carA, and ptsI experiments. This file (.pdf) contains a figure showing the read depth coverage (y-axis) plotted against the genome position (x-axis) for flask 1 population samples for A. cysK, B. carA, C. and ptsI experiments. (PDF 316 kb) [file 12918_2018_653_MOESM7_ESM.pdf]

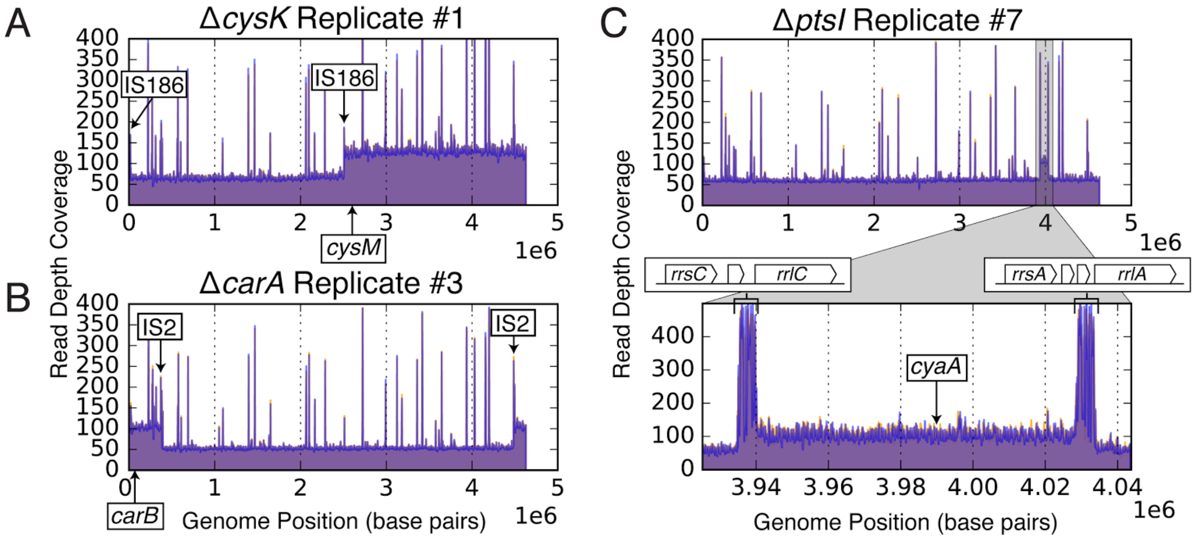

Additional File 7: Genome duplication amplification events observed in cysK, carA, and ptsI experiments. Read depth coverage (y-axis) is plotted against the genome position (x-axis) for flask 1 population samples for A. cysK, B. carA, C. and ptsI experiments. For cysK and carA samples (on the left), the regions of amplification are flanked by IS elements, IS186 and IS2, respectively. These regions of amplification contain the genes (cysM and carB) associated with a model-predicted alternate isozyme (cysM) or a previously reported multi-copy suppressor (carB). For the ptsI experiment (on the right), a smaller region is amplified and this region is magnified in the bottom plot. This region of amplification is flanked by ribosomal RNA genes and the identified metabolic gene of interest within this region is cyaA.
