# Supplementary material for: Reframing gene essentiality in terms of adaptive flexibility
Source: BMC Syst Biol. 2018 Dec 17;12:143. doi: 10.1186/s12918-018-0653-z (PMC6296033; doi:10.1186/s12918-018-0653-z)
Supplement: Supplementary file 8 — FBA simulations for genes listed in Table 1. This file (.pdf) contains a table of simulations demonstrating the same predicted behavior in both the iJO1366 model corresponding to the MG1655 genotype and a modified iJO1366 model corresponding to the BW25113 genotype.(PDF 79 kb) [file 12918_2018_653_MOESM8_ESM.pdf]

Additional File 8: FBA simulations for genes listed in Table 1. These simulations demonstrate the same predicted behavior in both the iJO1366 model corresponding to the MG1655 genotype and a modified iJO1366 model corresponding to the BW25113 genotype.

**Keio strain**	**Gene bnumber**	**Associated Reaction of interest (iJO1366 model ID)**	**Gene Reaction Rule**	**Isozyme or Alt. pathway reaction**	**Alt. Pathway Flux in BW25113 Model (modified iJO1366)**	**Alt. Pathway Flux in KO simulation BW25113 Model**	**Alt. Pathway Flux in iJO1366**	**Alt. Pathway Flux in KO simulation iJO1366 Model**
cysK	b2414	CYSS	b2414 or b2421	cysM	0.2395	0.2395	0.2395	0.2395
metL	b3940	ASPK; HSDy	b0002 or b3940 or b4024; b3940 or b0002	thrA or lysC	1.05; -0.6858	1.05; -0.6858	1.05; -0.6858	1.05; -0.6858
metC	b3008	CYSTL	b1622 or b3008	malY	0.1511	0.1511	0.1511	0.1511
thrA	b0002	ASPK; HSDy	b0002 or b3940 or b4024; b3940 or b0002	metL or lysC	1.05; -0.6858	1.05; -0.6858	1.05; -0.6858	1.05; -0.6858
carA	b0032	CBPS	b0032 and b0033	CBMKr	0.6155	0.6155	0.6155	0.6155
cysP	b2425	MOBDabcpp	(b0763 and b0764 and b0765) or (b2422 and b2424 and b2423 and b3917) or (b2422 and b2425 and b2424 and b2423)	(modA+modB+ modC)	0.0001246	0.0001246	0.0001246	0.0001246
proB	b0242	GLU5K	b0242	NACODA	0	0.217	0	0.217
ptsI	b2416	GLCptspp	(b2417 and b1621 and b2415 and b2416) or (b2417 and b1101 and b2415 and b2416) or (b1817 and b1818 and b1819 and b2415 and b2416)	GLCt2pp	0	10	0	10
serB	b4388	PSP_L	b4388	GHMT2r	1.096	-0.579	1.096	-0.579
proA	b0243	G5SD	b0243	NACODA	0	0.217	0	0.217
